# Deontology and Utilitarianism in Real Life: A Set of Moral Dilemmas Based on Historic Events

**DOI:** 10.1177/01461672221103058

**Published:** 2022-06-24

**Authors:** Anita Körner, Roland Deutsch

**Affiliations:** 1 University of Kassel, Germany; 2 University of Wuerzburg, Germany

**Keywords:** moral dilemma, deontology, utilitarianism, realism, plausibility

## Abstract

Moral dilemmas are frequently used to examine psychological processes that drive decisions between adhering to deontological norms and optimizing the outcome. However, commonly used dilemmas are generally unrealistic and confound moral principle and (in)action so that results obtained with these dilemmas might not generalize to other situations. In the present research, we introduce new dilemmas that are based on real-life events. In two studies (a European student sample and a North American MTurk sample, total *N* = 789), we show that the new factual dilemmas were perceived to be more realistic and less absurd than commonly used dilemmas. In addition, factual dilemmas induced higher participant engagement. From this, we draw the preliminary conclusion that factual dilemmas are more suitable for investigating moral cognition. Moreover, factual dilemmas can be used to examine the generalizability of previous results concerning action (vs. inaction) and concerning a wider range of deontological norms.

## Real-Life Dilemmas: Scenarios Contrasting Deontology and Utilitarianism Based on Historic Situations

Imagine being the head of the French Agency for biomedicine in 2007 and having to decide whether or not to introduce a new death criterion for organ donation in test hospitals. Currently, many people die because they do not receive a donor organ. Most people in France are organ donors, but most of the organs are not transplantable because of poor oxygen supply for too long. The suggested new criterion would increase the number of transplantable organs. However, in rare cases, people could be reanimated after meeting this new criterion. Thus, if you introduce this new criterion, some people will die who might have been saved. Still, introducing this new criterion would lead to many more people surviving by enabling more organ transplants. Is it appropriate to introduce this new criterion? This is a moral dilemma because every decision is morally wrong. Introducing the new criterion leads to withdrawing life-saving reanimation procedures—violating the deontological norm of saving human lives. However, not introducing the new criterion leads to more deaths—violating the utilitarian principle of optimizing the outcome.

Although moral dilemmas where deontological and utilitarian principles clash are often used to examine moral cognition, the employed dilemmas differ considerably from the example provided above. For example, in the footbridge dilemma (Thomson, 1976; building on Foot, 1967), a runaway trolley will shortly overrun five workmen who are on the tracks. Next to you on a footbridge over the tracks is a very large stranger. The only way to save the workmen is to push this stranger off the footbridge onto the tracks where his bulk would stop the trolley. The stranger would die but the workmen would be saved. Both the footbridge and the organ harvesting dilemma require deciding between an option that is right according to deontology and an option that is right according to utilitarianism. However, the footbridge dilemma feels unrealistic and to many people even absurd. This might lead to participants’ not taking dilemma judgments seriously. Accordingly, dilemmas used in morality research have been widely criticized (e.g., Bauman et al., 2014; Hofmann et al., 2014).

In the present work, we present a set of new moral dilemmas based on real-life situations, such as the organ harvesting situation described above. We suggest that these dilemmas are experienced as more realistic, which causes higher participant engagement. Dilemmas with high participant engagement, in turn, likely engage psychological processes that are more similar to moral judgments in real life. Accordingly, these *factual dilemmas* are more appropriate for examining moral cognition. In addition, factual dilemmas have other advantages over typically used dilemmas; they do not confound (in)action and moral principle, employ more varied situations, and increase the range of deontological norms that can be examined. By testing factual compared with other dilemmas, the present work is chiefly focused on improving the predominantly used method in moral dilemma research. As such, it does not aim at testing psychological theories. At the same time, we are convinced that this methodological contribution will improve future theory tests.

### Moral Dilemma Judgments

Dilemmas are used to examine decisions between adhering to moral norms and optimizing consequences. Conforming to the dominantly used terminology, we call judgments *deontological* if they favor the option prescribed by the philosophical principle of deontology (e.g., Kant, 1785/1998). *Deontology* requires acting in a way that is consistent with universal norms (e.g., do not kill) even if the consequences of the action involve suffering (Kant, 1785/1998). Thus, deontology forbids killing even if this refusal leads to the deaths of many more people. In contrast, we call a judgment *utilitarian* if it favors the option prescribed by the principle of utilitarianism (e.g., Mill, 1861/1998). *Utilitarianism* requires optimizing the overall well-being regardless of the nature of the action itself (Mill, 1861/1998). Thus, utilitarianism may require killing one person if it is the only means to save several people. We use these terms purely descriptively and for terminological coherence with previous research; we are not implying that deontological or utilitarian judgments are driven by participants’ philosophical considerations. Deontological compared with utilitarian judgments have also been conceptualized as favoring norms compared with consequences (Gawronski et al., 2017) and as action-based compared with outcome-based (Cushman, 2013). As the present work is methodological, we take an agnostic stance concerning these positions.

Deontological and utilitarian judgments can result from several psychological processes. For example, norm-based reasoning has been found to increase deontological judgments (Broeders et al., 2011; Körner & Volk, 2014); while manipulations that increase the outcome disparity between the two options have been found to increase utilitarian judgments (Cao et al., 2017; Kawai et al., 2014; Moore et al., 2008; Petrinovich et al., 1993). Thus, both concerns about norms and consequences influence moral dilemma judgments. In addition, psychological processes that are unrelated to the moral principles of deontology and utilitarianism have also been shown to influence moral judgments; most prominently, affective processes (Bartels & Pizarro, 2011; Gleichgerrcht & Young, 2013; Valdesolo & DeSteno, 2006), the amount of cognitive deliberation (Moore et al., 2008; Suter & Hertwig, 2011), and social evaluation and self-presentation processes (Everett et al., 2016; Rom & Conway, 2018).

However, the above-described results were obtained using selections from the same set of moral dilemmas (introduced by Greene et al., 2001; extended and modified by Moore et al., 2008; see also Christensen et al., 2014; Lotto et al., 2014). These *trolley-type dilemmas* are the predominantly used stimuli in moral dilemma research (e.g., Hauser et al., 2007; Laakasuo et al., 2017). Trolley-type dilemmas are based on a small set of highly stylized situations with many shared characteristics, for example, emergency situations and an identifiable victim who suffers as a result of the utilitarian option. This homogeneity of stimuli undermines stimulus sampling (Wells & Windschitl, 1999) and entails the risk of results being shaped by the specific characteristics of the stimulus set (see e.g., Bauman et al., 2014).

### Low Realism in Trolley-Type Dilemmas

Several trolley-type dilemmas were originally devised as philosophical thought experiments, meant to illustrate intuitions concerning morally relevant distinctions (e.g., Thomson, 1976). Accordingly, these dilemmas highlight contrasting moral principles in pure form, abstracted away from real-life situations. This high abstraction, however, entails low realism. Trolley-type dilemmas have been termed “outlandish” (Gold et al., 2014, p. 1), “far-fetched, and often convoluted” (Kahane, 2015, p. 515), “highly artificial” (Sauer, 2018, p. 151), “utterly strange” (Sauer, 2018, p. 152) and “freakish” (Fried, 2012, p. 511), “so impoverished as to be downright cartoonish” (Wood, 2011, p. 69). Thus, although trolley-type dilemmas are well-suited as philosophical thought experiments, their unrealistic nature can impair their suitability for examining moral cognition.

This very low realism is problematic for at least two reasons: noncomparability between the world portrayed in dilemmas and the real world and, relatedly, the engagement of different psychological processes. First, it has been argued that trolley-type dilemmas are so unrealistic as to presuppose a very different world from the world we know (e.g., Davis, 2012). For example, in dilemmas, all consequences of each possible action follow deterministically (e.g., torturing a suspect will definitely lead to an innocent victim’s being saved; whereas not torturing the suspect will definitely lead to the victim’s death), and the actor has complete knowledge of these consequences. However, as moral intuitions are developed in real life (e.g., where torture might do more harm than good), assessing them in situations where real-world aspects are stipulated to be absent may yield nonsense results. Consequently, decisions may not be transferable to moral situations that could occur in real life (Davis, 2012; Elster, 2011). Therefore, some scholars consider it doubtful what we can learn from answers to trolley-type dilemmas (see also Martena, 2018; Sauer, 2018; Woodward & Allman, 2007).

Second, trolley-type dilemmas may engage different processes compared with real-world dilemma situations. Although to date we know little about psychological processes during real-life dilemma judgments, amusement is certainly not prominent. Trolley dilemmas, in contrast, have been found to amuse participants (about one third of participants felt amused by the trolley dilemma and about two third by the footbridge dilemma; Bauman et al., 2014). Thus, low realism probably alters the psychological processes dominating moral judgments (see also Knutson et al., 2010).

In a theoretical model of narrative text processing, low realism has been postulated to negatively affect text processing (Busselle & Bilandzic, 2008). Specifically, the detection of realism violations is postulated to impair imagery concerning the narrative, thereby reducing participant engagement and consequently outcomes that depend on participant engagement, such as entertainment and persuasion (Busselle & Bilandzic, 2008; Busselle & Vierrether, 2022). Consistent with this theory, realism has been found to predict message evaluation so that higher realism was associated with more persuasion (Cho et al., 2014) and narrative-consistent thoughts (Green, 2004). Conversely, fictional stories with low realism have been found to yield no narrative persuasion (Nera et al., 2018). In sum, low (vs. high) realism adversely affects text processing by reducing engagement. In the present research, we partially test this theoretical prediction by examining whether dilemma realism is associated with participant engagement.

Empirically, scenarios are most frequently judged to be unrealistic if they are (a) implausible, (b) untypical for most people, or (c) not based on facts (Hall, 2003). Trolley-type dilemmas are typically low on all three realism criteria. First, these dilemmas are usually not based on facts but are purely hypothetical. Second, decisions that directly and immediately result in life or death of other human beings are very seldom and that these decisions involve out-of-control trains or other fanciful emergencies is even more rare. Thus, the situations are certainly anything but typical events in most people’s lives. Third, many trolley-type dilemmas consist of implausible options; both the range of options and these options’ alleged consequences contradict most readers’ knowledge about the world. Specifically, in many dilemmas, it seems implausible that a group will certainly die when a specific action is not performed (e.g., five people will be overrun by a nearing trolley: no one will notice the trolley in time, no one will survive the contact) but that the whole group will be saved if the suggested action is performed (one heavy man will stop the trolley: pushing him will have him falling on the tracks, and his weight is sufficient to stop the trolley that would otherwise kill five).

Plausibility has been found to systematically influence dilemma judgments (Körner et al., 2019). If a dilemma’s range of options and these options’ consequences were implausible (vs. plausible), participants were less willing to transgress deontological moral norms. Thus, low plausibility led to comparatively deontological judgments (Körner et al., 2019; for similar results, see Kneer & Hannikainen, 2022). As dilemmas have no correct answer, it is not clear whether the relatively utilitarian judgments in the plausible dilemma versions are less biased than the relatively deontological judgments in implausible versions. However, as plausibility, a central aspect of realism, influences moral judgments, this indicates that participants are influenced by low realism instead of ignoring it. Thus, dilemma judgments may be distorted by variations in realism. The association between realism and dilemma judgments will be further examined in the present research.

### Additional Problems With Trolley-Type Dilemmas

In addition to low realism, trolley-type dilemmas have been criticized for confounding moral principle and action compared with inaction (Gawronski & Beer, 2017; van den Bos et al., 2011; see also Manfrinati et al., 2013; Miller et al., 2014; Patil, 2015). Specifically, in trolley-type dilemmas, the deontological option is always an inaction while the utilitarian option always involves active interference. In the footbridge dilemma, for example, the deontological option means not interfering, letting the trolley proceed and overrun five people, whereas the utilitarian option requires actively pushing a stranger onto the tracks so that his bulk stops the trolley. Time pressure, for example, might alter either moral processes or preference for inaction—both could lead to the same judgments. Thus, using only dilemmas where moral principle and action/inaction are confounded, one cannot distinguish between processes that affect inaction tendencies and processes that affect utilitarian compared with deontological tendencies (for an approach to disentangle these determinants, see Gawronski et al., 2017). Note, however, that preferences for action/inaction need not necessarily be unrelated to morality. Regarding harm caused by commission (breaking proscriptive norms) as more severe than harm caused by omission (breaking prescriptive norms) can be genuinely moral (Janoff-Bulman et al., 2009; cf. Spranca et al., 1991) Thus, processes related to (in)action may be genuinely moral processes.

Finally, trolley-type dilemmas are problematic because of their strong predominance in the field. Many highly influential studies use only trolley dilemma variations (e.g., Awad et al., 2020; Greene et al., 2009; Hauser et al., 2007; Valdesolo & DeSteno, 2006). This predominance induces other scientists to adhere to this de facto standard. Barak-Corren and Bazerman (2017, p. 284), for example, justified their decision to use the trolley dilemma by stating “our use of the trolley problem is not motivated by any great interest in railway ethics, and there are disadvantages . . . [T]he trolley problem has been extensively studied, and our experimental approach and hypotheses draw extensively upon the specific lessons of this existing literature.” Thus, by now, trolley dilemmas are so extensively used that more researchers feel pressured to use them. A common method within a field of research is useful for comparing results and building a coherent body of knowledge. However, when results are only demonstrated with one method, it is unclear to which situations these results can be generalized (Wells & Windschitl, 1999). Accordingly, the strong reliance on a small set of dilemmas has raised doubts about the generalizability of moral judgment results (Bauman et al., 2014).

In sum, these arguments—trolley dilemmas are being perceived as unrealistic and they confound moral principle and (in)action—have raised concerns about the validity of trolley-type dilemmas for examining moral cognition. In addition, their strong predominance has raised doubts about the generalizability of established findings beyond trolley-type dilemmas. Thus, expanding and improving the set of dilemmas is a worthwhile goal for moral cognition research. There have been previous attempts at using more realistic dilemmas (e.g., Baron et al., 2015; Kahane et al., 2012; Piazza & Landy, 2013; Takamatsu, 2019). However, these dilemmas were generally created on the spot to examine specific questions. Accordingly, each publication contained only a small number of dilemmas and these dilemmas were usually not extensively pretested. In the present research, we reverse the focus; instead of testing theoretically derived predictions about moral judgments, we focus on examining whether the present new dilemmas are seen as more realistic and lead to higher participant engagement than trolley-type dilemmas.

### The Present Factual Dilemmas

In the present research, we introduce a set of new dilemmas. These factual dilemmas are created as stimuli for moral psychology experiments where trolley-type dilemmas predominate. The basic requirement for these types of dilemmas is that they describe situations where a decision has to be made between two mutually exclusive options—one consistent with deontology and the other consistent with utilitarianism. Accordingly, our goal was to keep this conflict between deontology and utilitarianism as clear as possible. The new features compared with trolley-type dilemmas were (a) increased realism, which should facilitate participant engagement (Busselle & Bilandzic, 2008); (b) more situational variety (e.g., not only emergency situations) as well as more varied norms; and (c) removal of the confound between (in)action and moral principle.

A main advantage of the present factual dilemmas is that all dilemmas are based on real-life occurrences. By describing events that did occur, they fulfill the realism criterion of factuality. As they contain specific details, such as place and date, we expect that their factual nature is apparent to readers. We also hypothesize that factual (vs. trolley-type) dilemmas are perceived to be more plausible and more typical of real-life events.

Thematically, factual dilemmas involve predominantly medical decisions, emergencies resulting from accidents, and situations arising through war, terrorism, or crime. For an overview, see Tables 1 and 2; for full texts, see the Appendix. Structurally, factual dilemmas fall into three distinct classes. First, a number of factual dilemmas are similar to trolley-type dilemmas in that they describe a decision whether or not to kill someone to save more lives. The main differences are that factual–killing dilemmas are more realistic and include a wider range of situations as well as information about the general setting and the people involved.

**Table 1.** Content Information About Factual Dilemmas Involving Killing or Saving.

No	Dilemma name	Context	Victims of utilitarian choice	Benefited people of utilitarian choice (in life-or-death situations: saved)	Suffering subset of benefited?^a^	Self in benefited group?	Feels outdated?	Opposite framing reasonable?^b^
Factual–Killing (action coincides with utilitarian option)								
1	Conjoined Twins	Medicine	One’s infant daughter	The other infant twin daughter	Yes	No	No	Not really
2	Rope ladder	Accident	An unknown man	Unspecified number of unknown people	Probably no	Yes	No	No
3	Cannibalism on Lifeboat	Accident	A 17-year old ship boy (younger than oneself and of lower rank)	3 men	Yes	Yes	Yes	No
4	Doodlebug	War	An unknown number of people from the lower class	Unspecified (greater) number of unknown people	Probably no	Probably no	Yes	Not really
5	Plague spread	Medicine	Unknown number of one’s parishioners	Unspecified (greater) number of unknown people	No	No, self potentially in victim-group	Yes	No
6	Euthanasia (in ghetto)	War	Large number of infants and children	The same individuals (more quick and painless death)	Yes	No	Yes	No
7	Immunization (smallpox)	Medicine	One’s gardener’s son	Unspecified number of unknown people	Probably no	No	Yes	No
8	911	Terrorism	Self and all people on same plane	Unspecified number of unknown people	Yes	No, self in victim-group	Rather no	Not really
9	Coma (mother)	Medicine	One’s own mother	Self and relatives	No	Yes	No	No
10	Organ donation law	Medicine	Unspecified number of unknown people	Unspecified (greater) number of unknown people	Probably no	No	No	Yes
11	Still births	War	Specific shortly-to-be-born babies	The babies’ mothers	Yes	No	Yes	Not really
12	Tyrannicide	War/ Terrorism	The dictator	Unspecified (greater) number of unknown people	No	Maybe	Rather no	No
13	Real lifeboat	Accident	10 people in lifeboat	The remaining passengers	Yes	Yes	Yes	No
14	Septuplets	Medicine	3 (random) fetuses	4 remaining fetuses	Yes	No	No	No
15	Assisted suicide state	Medicine	Unspecified number of unknown people	Unspecified (greater) number of unknown people	No	No	No	Yes
16	Atom bomb	War/ Terrorism	Unspecified number of unknown people	Unspecified (greater) number of unknown people	No	Not likely	Yes	Not really
Factual–Saving (action coincides with deontological option)								
17	Medicine costs	Medicine	A 7-year-old boy	Unspecified (greater) number of unknown people	No	No	No	Rather yes
18	RAF kidnapping	Crime/Terrorism	The President of the German employers’ organization	Unspecified (greater) number of unknown people	No	No	Rather yes	Yes
19	Mare nostrum	Humanitarian Aid	Unspecified number of unknown people	Unspecified (greater) number of unknown people	No	No	No^c^	Yes
20	Rwanda	Crime/ Terrorism	One’s employee	Family of another employee + self (+ maybe others)	Yes	Yes	No	Yes
21	(Sahara) Ransom	Crime/ Terrorism	10 specific people (not personally known to oneself)	Unspecified (greater) number of unknown people	No	No	No	Yes
22	Aid for Syria	Humanitarian Aid	Unspecified number of unknown people	Unspecified (greater) number of unknown people	Maybe	No	No	Yes

a Suffering subset of benefited: describes whether the victims of the utilitarian choice also belong to the group of people who suffer when a deontological choice is made. In factual-killing dilemmas, this is death avoidability (will the victim die in either case, see e.g., Moore et al., 2008). ^b^ Opposite framing reasonable: Can the question be framed as to make other principle (deontology or utilitarianism) coincide with active interference (without substantial dilemma modifications). ^c^ Unless one considers that GB is no longer in EU.

**Table 2.** Content Information About Factual Dilemmas Involving Other Norms.

No	Dilemma name	Context	Norm transgression	Victim (=sacrifice) of utilitarian choice	Characteristics of benefited people (of utilitarian choice)	Victim subset of benefited?^a^	Self in benefited group?	Feels outdated?	Opposite framing reasonable?^b^
Factual Other proscriptive norms (action coincides with utilitarian option)									
23	Rescue torture	State authority/ Justice	Torture	A confessed child-kidnapper	A kidnapped boy	No	No	No	No
24	War torture	War	Torture	An unknown number of war prisoners	Your compatriots (probably)	Probably no	Not likely	Yes	No
25	Rugby Cannibalism	Accident	Eating bodies	Dead bodies of teammates	Survivors	No	Yes	Rather yes	No
26	Robin Hood	Crime	Abusing trust & committing fraud	Trust and interest of some of one’s clients	One’s indebted clients	No	No	No	No
27	Knee operations	Medicine	Bodily inviolateness	2 people with knee problems	Unspecified number of unknown people	No	No	No	No
28	Organ market	Medicine	Bodily inviolateness & abusing poverty	Unknown person from poor country	Self	No	Yes	No	Not really
29	Tax fraud detection	Justice	Making use of & rewarding crime	The law (+credibility?)	State finance	?	State and its citizens	No	somewhat
30	Ashley treatment	Medicine	Bodily inviolateness	A handicapped girl (her rights and body)	The girl and her parents	Yes but will also benefit	No	No	Yes
31	Test tube sibling	Medicine	Creating a child as an organ donor	A planned baby	A small boy	No	No	No	Yes
32	Lion hunt	Animal rights	Animal deaths	A few lions	The majority of lions	Yes	No	No	No
Factual Other prescriptive norms (action coincides with deontological option)									
33	Bishops	War	Not speaking up against genocide	Missed opportunity of giving moral support to threatened group	Unspecified number of people, mostly unknown	No	No	Yes	Somewhat
34	Trail of tears	Justice	Not standing up for one’s tribes’ rights	One’s tribe (giving up the land)	Unspecified number of one’s tribe members	Yes	Yes, plus self potentially in victim group	Yes	Yes
35	Veterinarian	Animal rights	Animal suffering	Animals in one’s lab	Unspecified number of unknown animals	No	No	No	Yes
36	Hogesa (hooligan demo)	State authority	Disregarding freedom of assembly (for extremist group)	Right-wing group	Health of potentially many people	No	No	No	Yes
37	Mass rape	Justice	Punishing innocent	1 (unknown) of 8 specific men	Unknown number of unknown people	No	No	Rather no	Yes
38	Endowment	Justice	Mockery and abuse of crime victim (according to family)	Memory of victim + family	Children who were crime victims	No	No	No	Yes

a Suffering subset of benefited: describes whether the victims of the utilitarian choice also belong to the group of people who suffer when a deontological choice is made. In factual-killing dilemmas this is death avoidability (will victim die in either case, see, for example, Moore et al., 2008). ^b^ Opposite framing reasonable: Can question be framed as to make other principle (deontology or utilitarianism) coincide with active interference (without substantial dilemma modifications).

Second, in some factual dilemmas, the pairing of action (vs. inaction) with utilitarianism (vs. deontology) is reversed. As already mentioned, trolley-type dilemmas always employ proscriptive deontological norms, that is, forbidding specific actions. Some factual dilemmas employ prescriptive deontological norms, that is, norms that involve actions, such as saving human lives. Studies using dilemmas where action coincides with deontology can show whether judgments in dilemmas with proscriptive and prescriptive norms employ similar psychological processes.

Third, factual dilemmas employ a wider set of deontological moral norms. Trolley-type dilemmas always involve the question of whether or not it is appropriate to kill a human being, with torture as the only exception. In addition to 22 life-or-death situations (decisions about killing or saving lives), the factual dilemmas contain 16 dilemmas involving other deontological moral norms, such as norms about bodily inviolateness, animal suffering, or intentionally breaking laws (for a complete list, see Table 2). These dilemmas can be used to test whether an influence on dilemma judgments generalizes beyond killing/saving decisions to other moral dilemma judgments. In sum, in addition to increasing realism, the factual dilemmas allow for three steps of increasing generalization compared with trolley-type dilemmas, from more varied and detailed but structurally similar dilemmas, over a generalization concerning action/inaction, to general dilemma situations (i.e., beyond killing/saving) contrasting consequence optimization with the adherence to commonly endorsed deontological moral norms.

Here, we present two studies where participants evaluated trolley-type and our newly developed factual dilemmas. To determine whether factual dilemmas are superior to trolley-type dilemmas as stimuli for moral psychology studies, we used perceived realism as a first criterion. Trolley-type dilemmas are generally perceived to be unrealistic, which could distort results (see above). Moreover, realism has been found to influence dilemma judgments (Kneer & Hannikainen, 2022; Körner et al., 2019). Therefore, we consider highly realistic dilemmas to be superior as stimuli for moral psychology. Accordingly, we asked participants in both studies to evaluate dilemma realism. As a second quality criterion, participant engagement was assessed. High participant engagement is a precondition of dilemma judgment validity (Bauman et al., 2014). Moreover, higher realism should increase participant engagement (Busselle & Bilandzic, 2008). To measure participant engagement, we assessed response times (Study 1) and participants’ judgments how seriously they took the dilemmas (Study 2). We predicted that factual dilemmas would be perceived to be more realistic than trolley-type dilemmas and would lead to higher participant engagement.

## Study 1

We developed dilemmas based on historic facts. For this, we gathered information on various events where norms and consequences seemed incommensurate. We excluded situations for which viable alternative actions (compared with both the default and the intervention option presented in the dilemma scenario) seemed possible (see Körner et al., 2019) and situations for which we did not find at least one source reporting the main facts in a reputable journalistic or historic/scientific outlet. Using the remaining events, we wrote dilemma texts, highlighting the situation, the options, and their consequences in a manner similar to trolley-type dilemmas. However, the descriptions were generally longer and contained more details.

The main aim of Study 1 was to test how these factual dilemmas are evaluated compared with trolley-type dilemmas. Our hypothesis was that factual dilemmas would be perceived to be more realistic than trolley-type dilemmas—more typical of real-life situations, more plausible, and less absurd. Moreover, if higher realism increases participant engagement, we would expect participants to spend more time on reading and answering factual (vs. trolley-type) dilemmas.

### Method

To prevent fatigue-related effects, the dilemmas were split into smaller subsets of nine to 20 dilemmas, comprising six sets, each used in one data collection wave.^1^ In both studies, all measures, manipulations, and exclusions are reported. Neither study was preregistered. All stimuli, materials, raw data, and analysis files can be found at: https://osf.io/cg5tq/.

#### Participants

A total of 545 German-speaking participants (406 female, 139 male, *M*_age_ = 27 years, *SD*_age_ = 9) from the local participant pool (consisting mainly of students from different disciplines) completed the study in the lab, approximately equally distributed across the data collection waves, in exchange for 9€/hour.^2^

We expected at least medium-sized effects when comparing factual and trolley-type dilemmas for realism. However, to gain more precise estimates (and be able to compare individual dilemmas), we employed a much larger sample size, at least 80 participants per dilemma. The final sample size depended on logistic constraints. With 545 participants and α = .05, the sensitivity analysis for Study 1 yields 80% power to detect, *d*_z_ = 0.12.

#### Dilemmas

As factual dilemmas, we used 16 killing dilemmas (similar to trolley-type dilemmas), six saving dilemmas (using the opposite moral principle–action pairing), and 16 dilemmas involving other norms (10 of which use a utilitarianism–action pairing and 6 a deontology–action pairing). For an overview of the content, see Tables 1 and 2, and for full texts (German and English), see the Appendix.

For comparison, we selected 15 frequently used trolley-type dilemmas from Moore et al. (2008) and Greene et al. (2001): *Lifeboat, Fumes, Safari, Vitamins, Transplant, Lawrence of Arabia, Vaccine Test, Submarine, Plane Crash, Burning Building, Preventing the Spread*, and *Rescue 911*, as well as the most frequently used trolley-type dilemmas: *Trolley, Footbridge*, and *Crying Baby*.

#### Measures

The moral judgment question was *How appropriate is it to [perform suggested action] to [achieve goal]*? Participants answered on a 7-point scale, ranging from 1 (*not at all appropriate*) to 7 (*completely appropriate*).

Then participants were asked to evaluate their judgment and the dilemma on several 7-point scales. First, using the questions from Christensen et al. (2014), participants evaluated their feelings during the moral judgment, specifically experienced valence (1 = *very negative*; 7 = *very positive*) and arousal (1 = *not at all arousing*; 7 = *very arousing*). Next they rated the judgment difficulty (1 = *not at all difficult*; 7 = *very difficult*).

For dilemma realism, participants evaluated absurdness, typicality, and plausibility. Specifically, they rated the dilemma concerning absurdness (1 = *not at all absurd*; 7 = *very absurd*). Then, they indicated their agreement with four statements about dilemma typicality (adapted from Bauman et al., 2014), which were averaged into a typicality score. *The scenario is realistic. The scenario is similar to decisions that people have to make in real life. This would never happen in real life.* (reverse coded) *The scenario resembles real moral predicaments.* Then, plausibility was assessed as agreement with two statements, which were averaged into a plausibility score (Körner et al., 2019, Experiment 1). *It is probable that the described actions would have the stated consequences. It is plausible that, given the circumstances, there are* **no** *other options; that is, no reasonable action to achieve a better outcome.* Both typicality and plausibility were answered using 7-point scales (1 = *do not agree at all*; 7 = *agree completely*).

For the latter three (of six) data collection waves, participants were asked for each scenario (filler, trolley-type, and factual) whether, if the dilemma had been based on real-life events, they remembered hearing about it (by choosing one of the following four options, *no; it seems familiar but I have no specific recollections; I can remember the scenario but not what was decided; I can remember the scenario and its aftermath)*. Finally, participants were given the option to comment on the dilemma.

#### Procedure

Participants were informed that they would be reading and evaluating descriptions of dilemma situations, some of which would be based on historic events (that is, they had occurred in real life) while others were purely hypothetical. After providing informed consent, participants first read and evaluated a filler scenario^3^ to get familiar with the questions. Then they evaluated trolley-type dilemmas and factual dilemmas in random order with the restriction that not too many dilemmas of the same type succeeded each other, interspersed with a few filler scenarios. Participants were not informed to which kind (filler, factual, or trolley-type) any scenario belonged. After answering the moral judgment question, evaluating their feeling during the judgment, and evaluating the dilemma for realism and familiarity, the next dilemma ensued. After participants had evaluated all dilemmas, they provided demographic information and were given the chance to comment on the study.

### Results and Discussion

For each participant, mean scores for all judgment dimensions were calculated separately for factual and trolley-type dilemmas. Response times were divided by the number of words before means for each dilemma type were calculated. Factual dilemmas were judged to be more typical (*M* = 5.03, *SD* = 0.81) than trolley-type dilemmas (*M* = 3.90, *SD* = 1.03), *t*(544) = 30.56, *p* < .001, *d*_z_ = 1.31, 95% confidence interval (CI): [1.19,1.42]. Moreover, factual dilemmas were also judged to be more plausible (*M* = 4.18, *SD* = 0.85) than trolley-type dilemmas (*M* = 3.53, *SD* = 1.04), *t*(544) = 16.62, *p* < .001, *d*_z_ = 0.71, 95% CI [0.62, 0.81], and factual dilemmas were judged to be less absurd (*M* = 3.15, *SD* = 1.08) than trolley-type dilemmas (*M* = 4.73, *SD* = 1.19), *t*(544) = 33.28, *p* < .001, *d*_z_ = 1.43, 95% CI [1.31, 1.54].^4^ In sum, factual dilemmas were perceived to be substantially more realistic than trolley-type dilemmas with medium to large effects (an improvement of about one scale point on a 7-point scale). In addition, participants took longer to answer factual dilemmas (*M* = 274 ms, *SD* = 97 per word) than trolley-type dilemmas (*M* =250 ms, *SD* = 92 per word), *t*(544) = 9.85, *p* < .001, *d*_z_ = 0.42, 95% CI [0.33, 0.51], which speaks for greater engagement with factual than trolley-type dilemmas.

Tables 3 and 4 depict all evaluations on the dilemma level. As can be seen, dilemma evaluations vary substantially, both for trolley-type dilemmas and for factual dilemmas. Among trolley-type dilemmas, *Footbridge* was perceived to be particularly unrealistic whereas *Crying Baby* and *Lifeboat* were generally perceived to be comparatively realistic. Among factual dilemmas, historically very distant situations were generally perceived to be less realistic than more current dilemmas. Table 5 depicts the means for the different subclasses of dilemmas separately. Although judgment, valence, arousal, and difficulty did not significantly differ depending on dilemma type, there seem to be nonsignificant tendencies indicating that dilemmas involving other norms compared to life-or-death might be judged less arousing and less difficult. Table 6 shows exploratory correlations between moral judgment and dilemma evaluations as well as all dilemma evaluations among each other. These results indicate that all realism aspects—typicality, plausibility, and absurdness—correlated with moral judgments. Specifically, the higher the perceived realism of a dilemma, the more participants were willing to endorse the suggested action (for the same results separately for each dilemma type, see the Supplemental Materials). This finding is consistent with previous results (Körner et al., 2019), indicating that high plausibility increases utilitarian judgments.

**Table 3.** Mean Evaluations of Trolley-Type Dilemmas and Mean Dilemma Judgments in Study 1.

Dilemma name	Typicality	Plausibility	Absurdness	Appropriateness judgment	Arousal	Difficulty	Valence
Trolley	3.96	3.95	4.60	4.01	4.88	4.88	2.25
Footbridge	3.08	2.59	5.90	2.26	4.58	3.41	2.33
Crying Baby	4.68	4.05	3.66	3.36	5.69	5.17	1.51
Lifeboat	4.61	4.22	3.80	3.92	5.23	4.85	2.22
Fumes	3.70	3.28	5.02	3.92	5.10	4.86	2.15
Safari	3.92	3.87	4.76	3.70	5.33	5.20	1.99
Vitamins	3.23	3.05	5.70	2.92	5.03	3.69	2.39
Transplant	4.50	3.81	4.00	1.55	4.94	2.88	2.02
Plane Crash	3.82	3.60	4.99	2.24	5.04	3.48	1.75
Lawrence of Arabia	3.75	3.93	4.58	4.27	4.47	4.32	2.62
Vaccine Test	3.31	3.45	5.47	2.62	4.76	4.19	2.33
Submarine	4.13	3.95	4.43	4.98	4.77	4.71	2.31
Burning building	4.04	3.87	4.27	4.51	5.02	4.99	2.04
Preventing the Spread	3.77	3.30	4.74	4.92	4.29	2.80	3.06
Rescue 911	4.09	3.76	4.31	3.93	5.29	5.39	2.11

*Note.* Typicality, plausibility, and absurdness: 1 = *not at all*, 7 = *completely*; Appropriateness Judgment: 1= *not at all appropriate*; 7 = *completely appropriate*; Arousal: 1 = *not at all arousing*, 7 = *very arousing*; Difficulty: 1 = *not difficult*, 7 = *very difficult*; Valence: 1 = *very negative feelings*, 7 = *very positive feelings*.

**Table 4.** Evaluations and Judgments Concerning Factual Dilemma in Study 1.

No	Dilemma name	Typicality	Plausibility	Absurdness	Appropriateness judgment	Utilitarian judgment	Arousal	Difficulty	Valence	Familiarity
1	Conjoined Twins	5.80	5.65	2.37	5.45	5.45	5.26	4.60	2.21	2%
2	Rope Ladder	4.72	4.03	3.81	4.58	4.58	5.28	4.50	2.08	0%
3	Cannibalism on Lifeboat	4.46	4.09	3.85	2.95	2.95	5.20	4.01	2.01	8%
4	Doodlebugs	5.20	4.29	2.74	4.70	4.70	4.61	4.29	2.60	3%
5	Plague Spread	5.19	4.34	2.82	4.90	4.90	4.40	4.27	2.47	4%
6	Euthanasia (in Ghetto)	5.38	4.57	2.53	3.54	3.54	5.60	4.97	1.63	5%
7	Immunization (Smallpox)	4.80	3.86	3.33	2.89	2.89	4.68	3.72	2.62	2%
8	911	4.72	4.40	3.26	5.22	5.22	4.91	4.40	2.13	
9	Coma (Mother)	5.32	3.86	3.38	2.45	2.45	5.79	3.48	1.71	
10	Organ Law	4.98	4.25	3.45	2.97	2.97	4.94	3.77	2.66	
11	Stillbirths	4.46	4.24	3.69	3.87	3.87	5.70	5.17	1.71	
12	Tyrannicide	4.73	4.24	3.49	5.01	5.01	4.54	3.91	2.92	
13	Real Lifeboat	4.61	4.38	3.74	2.54	2.54	4.80	4.29	2.01	
14	Septuplets	4.72	4.49	3.96	4.11	4.11	4.96	4.65	2.12	
15	Assisted Suicide State	5.70	4.45	1.85	5.16	5.16	3.83	3.24	3.75	1%
16	Atom Bomb	5.22	3.97	2.78	3.59	3.59	4.50	4.28	2.50	19%
17	Medicine Costs	4.75	3.49	3.80	3.67	4.33	4.62	4.49	2.62	0%
18	RAF kidnapping	5.22	4.35	3.03	2.84	5.16	4.82	4.32	2.38	
19	Mare Nostrum	5.69	4.17	2.47	4.44	3.56	5.06	4.86	2.44	
20	Rwanda	4.77	4.00	3.60	4.80	3.20	5.14	4.87	2.47	
21	(Sahara) Ransom	5.47	4.54	2.56	3.92	4.08	4.71	4.71	2.25	
22	Aid for Syria	5.45	4.34	2.49	4.78	3.22	4.51	4.56	2.69	
23	Rescue Torture	5.55	4.38	2.43	4.20	4.20	4.72	3.97	2.70	22%
24	War Torture	5.07	3.95	3.11	3.28	3.28	5.05	4.48	2.28	
25	Rugby Cannibalism	4.55	4.78	3.86	4.67	4.67	5.30	3.82	1.92	
26	Test Tube Sibling	5.13	4.48	2.88	4.15	4.15	4.84	4.68	2.55	
27	Knee Operations	5.18	3.71	3.37	3.49	3.49	4.59	4.21	2.55	5%
28	Organ Market	5.82	4.81	2.13	3.67	3.67	4.71	3.83	2.46	6%
29	Tax Fraud Detection	5.00	4.46	2.78	4.72	4.72	3.03	3.14	3.67	
30	Robin Hood	4.74	3.79	3.63	2.29	2.29	3.91	3.25	3.09	
31	Ashley Treatment	4.73	3.82	3.96	2.81	2.81	4.74	4.05	2.36	
32	Lion Hunt	4.30	3.13	4.31	2.45	2.45	4.41	3.03	2.36	3%
33	Bishops	4.96	4.35	2.95	3.90	4.10	4.75	4.61	2.18	
34	Trail of Tears	4.62	4.23	3.15	2.97	5.03	4.39	3.57	2.60	
35	Veterinarian	5.01	4.11	3.15	4.41	3.59	4.59	3.77	2.65	
36	Hogesa (Hooligan Demo)	5.45	4.42	2.40	4.23	3.77	3.61	3.20	3.12	
37	Mass Rape	4.70	3.51	3.30	2.76	5.24	5.04	3.85	1.98	
38	Endowment	4.36	3.87	4.15	4.71	3.29	4.25	3.70	3.05	

*Note.* Typicality, plausibility, and absurdness: 1 = not at all, 7 = completely; Arousal: 1 = not at all arousing, 7 = very arousing; Valence: 1 = very negative feelings, 7 = very positive feelings; Decision difficulty: 1 = not difficult, 7 = very difficult; moral judgment: 1 = not at all appropriate, 7 = completely appropriate; utilitarian judgment: identical to appropriateness rating of action = utilitarian dilemmas; reverse coded for action = deontological dilemmas; Familiarity: percentage of participants who stated that they had heard about the situation and knew the outcome; missing values indicate that the question was omitted in the study batch containing this dilemma.

**Table 5.** Mean Evaluations for Each Dilemma Type in Study 1.

Dilemma type	Typicality	Plausibility	Absurdness	Judgment	Arousal	Valence	Difficulty
Trolley-type	3.91_a_	3.65_a_	4.68_a_	3.54_a_	4.96_a_	2.21_a_	4.32_ab_
Factual Killing	5.00_b_	4.32_b_	3.19_b_	3.99_a_	4.94_a_	2.32_ab_	4.22_ab_
Factual Saving	5.23_b_	4.15_b_	2.99_b_	4.07_a_	4.81_ab_	2.48_ab_	4.64_a_
Factual Other Norms with Action = Utilitarianism	5.01_b_	4.13_b_	3.25_b_	3.57_a_	4.53_b_	2.59_b_	3.85_b_
Factual Other Norms with Action = Deontology	4.85_b_	4.08_b_	3.18_b_	3.83_a_	4.44_b_	2.60_ab_	3.78_b_
ANOVA with Type as Independent variable	*F*(4, 48) = 18.03, *p* < .001	*F*(4, 48) = 4.91, *p* = .002	*F*(4, 48) = 15.24, *p* < .001	*F*(4, 48) = 0.71, *p* = .591	*F*(4, 48) = 2.38, *p* = .065	*F*(4, 48) = 1.68, *p* = .171	*F*(4, 48) = 2.35, *p* = .067

*Note.* Typicality, plausibility, and absurdness: 1 = *not at all*, 7 = *completely*; Judgment: 1 = *not at all appropriate*, 7 = *completely appropriate*; Arousal: 1 = *not at all arousing*, 7 = *very arousing*; Valence: 1 = *very negative feelings*, 7 = *very positive feelings*; Decision difficulty: 1 = *not difficult*, 7 = *very difficult*. For this table, means were calculated by averaging individual dilemma means (in the main analyses, means were calculated for each participant, and as participants answered only a subset of dilemmas, the means differ somewhat). Means that do not share subscripts within columns differ significantly, *p* < .05; for exact values, see https://osf.io/cg5tq/. ANOVA = analysis of variance.

**Table 6.** Correlation Matrix for All Measures (Treating Dilemma as the Unit of Analysis), Study 1.

Measure	Typicality	Plausibility	Absurdness	Arousal	Valence	Difficulty	Response time
Judgment	.289*	.491**	−.353**	−.171	.345*	.305*	.258
Typicality		.736**	−.969**	−.120	.217	.029	.236
Plausibility		—	−.755**	.024	.046	.237	.123
Absurdness			—	.209	−.286*	−.020	−.287*
Arousal				—	−.890**	.588**	−.085
Valence					—	−.506**	.179
Difficulty						—	.206

*Note.* Typicality, plausibility, and absurdness: 1 = *not at all*, 7 = *completely*; Judgment: 1 = *not at all appropriate*, 7 = *completely appropriate*; Arousal: 1 = *not at all arousing*, 7 = v*ery arousing*; Valence: 1 = *very negative feelings*, 7 = *very positive feelings*; Decision difficulty: 1 = *not difficult*, 7 = *very difficult*.

* *p* < .05. **p < .01.

## Study 2

Study 2 again compared factual dilemmas and trolley-type dilemmas. This time, we employed an online sample residing in the United States to examine whether the results from Study 1 generalize beyond German participants (which seemed especially important as several factual dilemmas occurred in Europe). Our hypothesis was that factual dilemmas would be again judged to be more realistic than trolley-type dilemmas. Moreover, as realism should influence participant engagement, we also hypothesized that participants would take factual dilemmas more seriously than trolley-type dilemmas.

### Method

The dilemmas were split into three subsets, so that each subset contained 22 dilemmas (2 identical filler scenarios, 7–8 trolley-type dilemmas, and 12–13 factual dilemmas). Each participant was randomly assigned to evaluate one subset of dilemmas.

#### Participants

A total of 244 participants (85 female, 157 male, 2 other gender, *Median*_age_ = 35 years) participated online via Amazon MTurk in exchange for $3.80.^5^ We again aimed for at least 80 participants per dilemma, which resulted in 80% power to detect, *d*_z_ = 0.18.

#### Dilemmas

We used the same trolley-type dilemmas as in Study 1 and, to have a similar ratio of factual and trolley-type dilemmas, added *Mine Shaft* and *Euthanasia* from Moore et al. (2008), and *Car Accident, Hard Times*, and *Boarder Crossing* from Conway and Gawronski (2013). To be able to examine whether there are any group differences between the three dilemma-subset conditions, every dilemma set included *Fumes*. Neither moral judgments nor any dilemma evaluations of *Fumes* differed between the dilemma subset conditions (see https://osf.io/cg5tq/). As factual dilemmas, we used the same dilemmas as in Study 1. They were translated to English and sometimes slight modifications were made.

#### Materials and procedure

The instructions were the same as in Study 1. After providing informed consent, participants first read and evaluated the *Heinz* dilemma, a filler scenario. For each scenario (filler, trolley-type, and factual), participants read the scenario and answered the moral judgment question *How appropriate is it to [perform suggested action]*?^6^ Participants answered on a 7-point scale, ranging from 1 (*completely inappropriate*) to 7 (*completely appropriate*).

Then participants were asked to evaluate their judgment and the dilemma on the same scales as in Study 1 with three changes. First, the scale anchors for the typicality and plausibility questions were changed to 1 = *strongly oppose*; 7 = *strongly support*. Second, the question about participants’ prior knowledge of the situation was modified. They indicated whether they thought the scenario was based on true facts and, if so, whether they remembered hearing about it (by choosing one of the following four options, *no, I do not think it happened; yes, I think it happened, but I do not remember hearing about it; yes, I remember the situation but not the results; yes, I remember the situation and also the results*). Third, participants indicated how seriously they took the scenario by indicating their agreement to *It was easy to take the scenario and the moral decision seriously* using a 7-point scale (1 = *strongly oppose*; 7 = *strongly support*). Except for these modifications, the materials and procedure were the same as in Study 1.

### Results and Discussion

For each participant, mean evaluation scores for each dimension were calculated separately for factual and trolley-type dilemmas. Factual dilemmas were judged to be more typical (*M* = 4.88, *SD* = 0.82) than trolley-type dilemmas (*M* = 4.33, *SD* = 1.05), *t*(243) = 9.64, *p* < .001, *d*_z_ = 0.62, 95% CI [0.48, 0.75]; factual dilemmas were judged to be more plausible (*M* = 5.02, *SD* = 0.85) than trolley-type dilemmas (*M* = 4.79, *SD* = 0.97), *t*(243) = 6.35, *p* < .001, *d*_z_ = 0.41, 95% CI [0.28, 0.54], and factual dilemmas were judged to be less absurd (*M* = 4.05, *SD* = 1.53) than trolley-type dilemmas (*M* = 4.68, *SD* = 1.34), *t*(243) = 10.08, *p* < .001, *d*_z_ = 0.65, 95% CI [0.51, 0.78], replicating the finding from Study 1 that factual dilemmas were judged to be more realistic than trolley-type dilemmas. Moreover, factual dilemmas were judged to be more easily taken seriously (*M* = 5.47, *SD* = 0.97) than trolley-type dilemmas (*M* = 5.02, *SD* = 1.18), *t*(243) = 8.84, *p* < .001, *d*_z_ = 0.57, 95% CI [0.43, 0.70], supporting our second hypothesis that factual compared with trolley-type dilemmas increase participant engagement.

Tables 7 and 8 depict evaluations on the dilemma level. Table 9 depicts the means for all evaluation dimensions for the different subclasses of dilemmas separately. This time, valence did differ significantly depending on dilemma type, with the least negative evaluations for dilemmas involving other norms compared with life-or-death decisions. Table 10 shows exploratory correlations among all judgments and dilemma evaluations. As in Study 1, the results indicate that all realism aspects—typicality, plausibility, and absurdness—correlated with moral judgments (for the same results separately for each dilemma type, see the Supplemental Materials). As in Study 1, higher realism was associated with more utilitarian judgments, indicating that realism affects morally relevant cognitive processes.

**Table 7.** Trolley-Type Dilemma Evaluations and Judgment in Study 2.

Dilemma	Typicality	Plausibility	Absurdness	Taking Seriously	Judgment	Arousal	Difficulty	Valence
Trolley	4.05	4.91	4.54	4.84	4.34	4.82	4.74	3.29
Footbridge	3.73	4.44	5.22	4.67	3.55	4.81	4.47	3.07
Crying Baby	4.35	4.81	4.55	5.01	3.93	5.45	5.28	3.03
Lifeboat	4.74	5.07	4.22	5.49	4.64	4.95	4.96	3.20
Fumes	4.06	4.80	4.90	4.86	4.41	5.01	5.17	3.34
Safari	4.29	4.84	4.88	5.11	4.40	5.31	5.34	3.50
Vitamins	3.88	4.28	5.21	4.43	3.57	4.84	4.38	3.32
Transplant	4.20	4.64	4.88	4.73	3.04	5.10	4.14	2.97
Plane Crash	4.32	4.61	4.76	4.89	3.55	5.05	4.20	3.08
Lawrence of Arabia	4.23	4.90	4.66	5.14	4.77	5.00	4.62	3.52
Vaccine Test	4.05	4.50	5.32	4.70	3.29	4.78	4.53	3.10
Submarine	4.49	5.04	4.34	5.29	4.81	4.90	4.97	3.16
Burning Building	4.54	5.00	4.30	5.26	4.96	5.00	4.86	3.24
Preventing the Spread	4.10	4.47	4.98	4.87	4.76	5.09	3.80	3.20
Rescue 911	4.03	4.65	4.89	4.81	4.18	5.23	5.19	3.46
Border Crossing	5.05	4.80	3.75	5.51	4.66	5.06	4.69	3.61
Car Accident	4.65	4.98	4.63	4.93	4.30	5.08	5.68	2.71
Euthanasia	4.89	5.27	4.12	5.56	4.41	5.47	5.12	2.86
Hard Times	4.87	4.61	4.95	5.33	2.88	5.04	4.27	2.73
Mine Shaft	4.55	4.97	4.44	5.16	4.59	5.21	5.24	3.53

*Note.* Typicality, plausibility, absurdness, taking seriously: 1 = *strongly oppose*, 7 = *strongly support*; Arousal: 1 = *not at all arousing*, 7 = *very arousing*; Valence: 1 = *very negative feelings*, 7 = *very positive feelings*; Decision difficulty: 1 = *not difficult*, 7 = *very difficult*; moral judgment: 1 = *completely inappropriate*, 7 = *completely appropriate*.

**Table 8.** Mean Evaluations and Judgments Concerning Factual Dilemmas in Study 2.

No	Dilemma name	Typicality	Plausibility	Absurdness	Taking seriously	Appropriateness Judgment	Utilitarian Judgment	Arousal	Difficulty	Valence	Familiarity
1	Conjoined Twins	5.16	5.34	3.74	5.64	5.13	5.13	5.31	5.24	3.45	16%
2	Rope Ladder	4.83	5.09	4.29	5.54	4.96	4.96	5.23	4.54	3.32	9%
3	Cannibalism on Lifeboat	4.40	5.00	4.77	5.23	3.82	3.82	5.04	4.74	2.75	11%
4	Doodlebugs	4.81	5.03	3.96	5.54	4.59	4.59	5.03	4.83	3.64	14%
5	Plague Spread	5.15	5.35	3.81	5.42	5.68	5.68	4.86	4.70	3.45	22%
6	Euthanasia (in Ghetto)	4.72	5.12	3.98	5.70	3.95	3.95	5.19	5.15	2.85	12%
7	Immunization (Smallpox)	4.71	4.68	4.21	5.33	4.34	4.34	4.86	4.80	3.63	13%
8	911	5.06	5.49	3.75	5.60	5.38	5.38	5.47	4.10	3.05	49%
9	Coma (Mother)	4.93	4.90	4.31	5.43	4.16	4.16	5.39	4.65	3.29	15%
10	Organ Law	4.73	4.77	4.10	5.27	3.79	3.79	4.91	4.43	3.46	11%
11	Stillbirths	4.31	5.12	4.96	5.18	4.52	4.52	5.10	5.75	2.82	11%
12	Tyrannicide	5.11	5.13	3.70	5.63	5.49	5.49	5.24	3.75	4.03	14%
13	Real Lifeboat	4.52	4.98	4.18	5.31	3.96	3.96	5.23	4.84	3.26	13%
14	Septuplets	5.04	5.12	3.95	5.68	5.03	5.03	4.86	4.69	3.22	11%
15	Assisted Suicide State	5.04	4.94	3.93	5.42	4.92	4.92	4.95	4.49	3.75	12%
16	Atom Bomb	5.24	5.09	3.52	5.63	5.10	5.10	5.03	4.49	3.64	29%
17	Medicine Costs	4.72	4.69	4.33	5.26	4.31	3.69	5.16	5.00	3.66	10%
18	RAF kidnapping	5.16	5.12	4.07	5.52	3.60	4.40	5.10	4.93	3.27	21%
19	Mare Nostrum	5.14	5.06	3.73	5.53	4.46	3.54	4.85	4.81	3.46	15%
20	Rwanda	4.88	5.12	4.01	5.64	4.62	3.38	5.11	5.40	3.21	11%
21	(Sahara) Ransom	5.31	5.34	3.57	5.73	4.46	3.54	5.20	5.23	3.47	9%
22	Aid for Syria	5.14	5.03	3.70	5.73	4.76	3.24	4.85	5.11	3.99	10%
23	Rescue Torture	4.91	5.11	3.96	5.61	4.85	4.85	5.21	4.38	3.84	8%
24	War Torture	5.04	5.17	3.92	5.56	4.42	4.42	5.23	4.59	3.49	14%
25	Rugby Cannibalism	4.81	5.35	4.20	5.38	5.13	5.13	5.09	4.57	3.08	33%
26	Test Tube Sibling	4.96	5.13	4.07	5.26	5.04	5.04	4.96	4.14	4.44	16%
27	Knee Operations	4.42	4.65	4.37	5.01	4.27	4.27	4.38	4.43	3.81	12%
28	Organ Market	4.98	5.12	4.06	5.50	4.74	4.74	5.03	4.75	3.96	6%
29	Tax Fraud Detection	4.90	4.82	4.04	5.41	4.15	4.15	4.52	3.82	4.11	14%
30	Robin Hood	4.63	4.84	4.29	5.49	3.88	3.88	4.86	4.33	3.96	13%
31	Ashley Treatment	4.65	4.71	4.38	5.40	4.16	4.16	4.64	4.86	3.57	13%
32	Lion Hunt	4.36	4.56	4.65	5.15	4.00	4.00	5.03	4.56	3.38	13%
33	Bishops	4.73	4.85	3.86	5.53	4.71	3.29	4.99	5.09	3.51	10%
34	Trail of Tears	5.22	5.36	3.48	5.82	4.29	3.71	5.27	4.93	3.22	25%
35	Veterinarian	5.28	4.99	3.74	5.84	4.92	3.08	5.04	4.64	3.93	12%
36	Hogesa (Hooligan Demo)	5.09	4.90	3.89	5.36	4.55	3.45	4.49	4.32	3.89	12%
37	Mass Rape	4.70	4.84	4.43	5.51	3.65	4.35	5.19	4.76	3.35	19%
38	Endowment	4.64	4.71	4.30	5.20	4.66	3.34	4.48	3.95	3.68	16%

*Note.* Typicality, plausibility, absurdness, taking seriously: 1 = *strongly oppose*, 7 = *strongly support*; arousal: 1 = *not at all arousing*, 7 = *very arousing*; Valence: 1 = *very negative feelings*, 7 = *very positive feelings*; Decision difficulty: 1 = *not difficult*, 7 = *very difficult*; moral judgment: 1 = *not at all appropriate*, 7 = *completely appropriate*; utilitarian judgment: identical to appropriateness rating of action = utilitarian dilemmas; reverse coded for action = deontological dilemmas; Familiarity: percentage of participants who stated that they had heard about the situation and knew the outcome.

**Table 9.** Mean Evaluations for Each Dilemma Type in Study 2.

Dilemma type	Typicality	Plausibility	Absurdness	Taking Seriously	Judgment	Arousal	Valence	Difficulty
Trolley-type	4.35_a_	4.78_a_	4.68_a_	5.03_a_	4.15^a^	5.06_ab_	3.20_a_	4.78_a_
Factual Killing	4.86_b_	5.07_b_	4.07_b_	5.47_b_	4.68^b^	5.11_a_	3.35_ab_	4.70_ab_
Factual Saving	5.06_b_	5.06_b_	3.90^b^	5.57^b^	4.37_ab_	5.04_ab_	3.51_bc_	5.08_a_
Factual Other Norms with Action = Utilitarianism	4.77_b_	4.95_ab_	4.19^b^	5.38^b^	4.47_ab_	4.89_b_	3.76_c_	4.44_b_
Factual Other Norms with Action = Deontology	4.94_b_	4.94_ab_	3.95^b^	5.54^b^	4.46_ab_	4.91_ab_	3.60_bc_	4.61_ab_
ANOVA with Type as Independent variable	*F*(4, 53) = 10.96, *p* < .001	*F*(4, 53) = 3.99, *p* = .007	*F*(4, 53) = 10.64, *p* < .001	*F*(4, 53) = 11.90, *p* < .001	*F*(4, 53) = 1.99, *p* = .109	*F*(4, 53) = 1.81, *p* = .142	*F*(4, 53) = 6.13, *p* < .001	*F*(4, 53) = 2.39, *p* = .062

*Note.* Typicality, plausibility, absurdness, taking seriously: 1 = strongly oppose, 7 = strongly support; Arousal: 1 = not at all arousing, 7 = very arousing; Valence: 1 = very negative feelings, 7 = very positive feelings; Decision difficulty: 1 = not difficult, 7 = very difficult; moral judgment: 1 = completely inappropriate, 7 = completely appropriate. For this table, means were calculated by averaging individual dilemma means (in the main analyses, means were calculated for each participant, and as participants answered only a subset of dilemmas, the means differ somewhat). Means that do not share subscripts within columns differ significantly, *p* < .05; for exact values, see https://osf.io/cg5tq/. ANOVA = analysis of variance.

**Table 10.** Correlation Matrix for All Evaluations (Treating Dilemma as the Unit of Analysis), Study 2.

Measure	Typicality	Plausibility	Absurdness	Arousal	Valence	Difficulty	Taking seriously
Judgment	.485^**^	.627^**^	−.600^**^	.086	.398^**^	.015	.484^**^
Typicality		.702^**^	−.904^**^	.137	.363^**^	−.019	.895^**^
Plausibility		—	−.716^**^	.409^**^	.021	.273^*^	.737^**^
Absurdness			—	−.095	−.450^**^	.003	−.874^**^
Arousal				—	−.360^**^	.341^**^	.233
Valence					—	−.349^**^	.280^*^
Difficulty						—	.102

*Note.* Typicality, plausibility, absurdness, taking seriously: 1 = *strongly oppose*, 7 = *strongly support*; Arousal: 1 = *not at all arousing*, 7 = *very arousing*; Valence: 1 = *very negative feelings*, 7 = *very positive feelings*; Difficulty: 1 = *not difficult*, 7 = *very difficult*; Moral judgment: 1 = *completely inappropriate*, 7 = *completely appropriate*.

* *p* < .05. ^**^p < .01.

## General Discussion

The majority of studies on moral dilemma judgments use a small set of dilemmas that have been widely criticized (e.g., Gigerenzer, 2010; Sauer, 2018). These trolley-type dilemmas are unrealistic, sometimes to the point of being ludicrous, they confound moral principle with (in)action, and they employ a small set of highly similar and impoverished scenarios. Low realism could distort moral judgments (Kneer & Hannikainen, 2022; Körner et al., 2019), and the strong predominance of homogeneous stimuli raises questions about the generalizability of empirical results (Woodward & Allman, 2007). In the present research, we presented and tested a set of new dilemmas based on historic events. These factual dilemmas were (a) perceived to be more realistic than the predominating trolley-type dilemmas; specifically, they were judged to consist of more plausible options, to be more similar to real-world moral quandaries, and to be less absurd. This higher perceived realism is probably why factual dilemmas led (b) to higher participant engagement; specifically, participants spent more time reading and deciding on factual compared with trolley-type dilemmas (Study 1) and were able to take them more seriously (Study 2).^7^ Therefore, if researchers intend that participants get deeply engaged with moral dilemmas, the present research provides initial evidence that this goal is more easily achieved with factual than trolley-type dilemmas.

In addition to testing the new, factual dilemmas, the present research also provides the first systematic realism assessment of frequently used trolley-type dilemmas. Our results indicate that trolley-type dilemmas vary in realism. While *Lifeboat, Crying Baby* (this one more in Europe than the United States), and *Border Crossing* were judged to be comparatively realistic, other dilemmas were judged unrealistic—especially the popular *Footbridge*. It is noteworthy that several studies contrast the footbridge dilemma with other trolley variations. Many of these find that an employed manipulation influences only judgments in the footbridge dilemma (e.g., Claessens et al., 2020; Costa et al., 2014; Duke & Bègue, 2015), concluding that the manipulation influences only judgments in up-close, personal dilemmas. Instead, the present results suggest that different judgments for *Footbridge* (vs. other dilemmas) might be related to its low realism. We advise researchers to employ only highly realistic dilemmas and especially advise against the usage of *Footbridge* as well as *Vitamins* and *Vaccine test*. Similarly, we also advise against one of our new dilemmas, *Lion Hunt*, as its realism evaluations are markedly inferior to the other factual dilemmas.

Compared with the German lab-based sample, the American MTurk sample showed smaller effect sizes, that is, smaller differences between factual and trolley-type dilemmas. This might have several reasons. First, the two samples differ in their average experiences with moral dilemmas. While the lab participants in Study 1 were comparatively unused to dilemma studies, many MTurk workers participate in a great number of studies (e.g., Chandler et al., 2014), and repeated experiences with dilemmas might dull one’s experiencing scenarios as unrealistic or absurd. This would explain the higher realism evaluations for trolley-type dilemmas in the MTurk (vs. lab) sample. Second, low motivation might lead to giving the same response to many questions. Overall, we observed a smaller response differentiation in the MTurk (vs. lab) sample (see Tables 3 and 6). And if there is less systematic variance, we would expect smaller effect sizes. Third, there might be cultural differences in what is deemed realistic. Differences in knowledge about the historic context might explain, for example, that the United States (but not the German) participants judged a dilemma about a Native American tribe fighting for its rights in 1830 more realistic than a dilemma about Nazi atrocities during World War II. Even for the majority of dilemmas that happened roughly in the present time, different laws and cultural practices might have influenced what is judged realistic. In general, when selecting dilemmas for experiments, realism judgments by a sample of participants with a similar background as the experimental participants should be considered.

Note that both the present participants and the factual dilemmas over-represent the Western world. Future research needs to determine whether these dilemmas are also judged to be realistic and lead to higher participant engagement than trolley-type dilemmas in other parts of the world. Moreover, adding more dilemmas that happened outside of Europe would probably increase realism for non-Western participants. In addition, future research should examine judgments in factual dilemmas by representative samples of participants.

### Validity Criteria for Moral Dilemmas

In the present research, we used realism and participant engagement as criteria to determine dilemma quality for moral psychology experiments. Using additional validation criteria would enable firmer conclusions. However, comparisons with previous findings are, in our opinion, not suitable to determine stimulus validity. These comparisons seem problematic because there is no way of knowing which findings are psychologically “correct” and which might be stimulus artifacts. Accordingly, neither finding similar effects for factual and trolley-type dilemmas nor finding different effects necessarily indicates validity.

Ultimately, the goal of any stimulus set should be to enable high internal and external validity. Concerning internal validity, stimuli need to be free from confounds (here, for example, concerning moral principle and action/inaction). In addition, dilemmas must depict a conflict between deontology and utilitarianism, for which, we argue, extraordinary situations are preferable to mundane situations. First, scenarios need unrecoverable losses to prevent participants from re-appraising the situation in a way that dissolves the dilemma. If bad things happen to a good person, participants tend to think that this person will be compensated (Harvey & Callan, 2014; see also Hafer & Rubel, 2015; Liu & Ditto, 2013). Many everyday sufferings, such as discomfort or monetary losses, could be compensated in the future, whereas severe suffering, especially death, cannot be compensated. This might explain why judgments concerning smaller stakes (monetary losses) have been found to yield different results from judgments concerning life-or-death decisions (Gold et al., 2014).

Second, in everyday life, decisions frequently involve many different options or uncertain consequences. However, as moral dilemmas need to clearly contrast deontology and utilitarianism, they require scenarios whose consequences occur with very high probability. For example, in the conjoined twin case, one baby would definitely die during the operation, and both babies would definitely die if the operation was not performed. In most real-life health care decisions, consequences are less certain. However, with uncertain consequences, one might gamble on making a decision that leads to optimal consequences without violating deontological principles (e.g., the survival of both babies). Thus, with uncertain consequences, the moral conflict task might, through re-appraisal, become an optimization task. In sum, for high internal validity, it is essential to have scenarios in which the consequences can be plausibly said to follow with high probability and entail unrecoverable losses, ensuring that deontology and utilitarianism do indeed require mutually exclusive actions.

External validity is the extent to which a study uncovers psychological processes that generalize across contexts and that play an important role in real-life situations (see Eastwick et al., 2013; Evans et al., 2015; Mook, 1983). As there is hardly any research on psychological processes in real-world dilemma situations, it is difficult to know which processes should be active in dilemma judgments. Accordingly, the external validity of moral dilemmas cannot be examined in the short run. A feasible alternative is a predictive validity. Predictive validity could be examined by testing whether judgments, first, predict participants’ own behavior or, second, predict how participants evaluate decisions made by others. First, in trolley-type dilemmas, judgments were found not to predict (or to predict only weakly) participants’ own behavior in similar situations (Bostyn et al., 2018). Thus, future research needs to determine whether factual dilemmas fare better at predicting participants’ behavior. Second, although individuals rarely have to decide on dilemmas themselves, they form public opinion and elect politicians who decide about dilemma-related topics, such as euthanasia, and behavior in health pandemics, war, and terrorist attacks. Thus, citizens frequently evaluate dilemmas at secondhand. Accordingly, examining whether factual (and trolley-type) dilemmas predict moral judgments at secondhand would be another valuable test of predictive validity.

### Usage of Factual Dilemmas

Factual dilemmas can be used instead of trolley-type dilemmas when examining psychological influences on dilemma judgments. Their novelty is in itself an advantage. When recruiting participants who might have participated in many studies before, using well-worn dilemmas could prevent participants’ forming new judgments. New dilemmas, in contrast, have to be evaluated on the spot and cannot be answered by memory retrieval. Besides being used in their present form, factual dilemmas can be modified. For example, when using them in combination with purely hypothetical dilemmas to enlarge the dilemma pool (i.e., without contrasting hypothetical and factual dilemmas), they may be shortened to make them more similar to trolley-type dilemmas (e.g., some of the present dilemmas, *Rwanda, Coma*, and *Bishops*, were modified to increase the number of dilemmas suitable for multinomial modeling, Körner et al., 2020). Note, however, that modifications could change perceived realism. Moreover, not only the dilemma texts but also the judgment question could be modified. For example, instead of evaluating how appropriate the focal action is, participants could be asked to judge whether the action is permissible, required, or signals a good moral character. Thereby, the factual dilemmas could be used to examine moral minimalism (Aktaş et al., 2017; Royzman et al., 2015) or moral character judgments (Uhlmann et al., 2013).

Factual dilemmas can be used to examine the generalizability of findings obtained with trolley-type dilemmas. First, factual-killing dilemmas may be used to examine whether any manipulation or process that was established with trolley-type dilemmas influences dilemma judgments also when realism is higher, the situations are more varied, and more context is available. Second, factual-saving dilemmas can be used to examine whether a manipulation or process influences dilemma judgments not only concerning proscriptive but also concerning prescriptive life-or-death decisions (for initial evidence, that realism might influence judgments in prescriptive and proscriptive dilemmas differently, see the Supplemental Figures S1 & S2). The final level of generalization concerns norms beyond life-or-death decisions. Factual dilemmas involving other norms (see Table 2) can be used to examine whether a manipulation or process influences dilemma judgments beyond killing/saving decisions. Thus, when selecting factual dilemmas, researchers can choose to examine dilemma judgments at differing degrees of generality.

In addition, some of the dilemmas can be easily reframed so that either the deontological or the utilitarian option is consistent with active interference (see Tables 1 and 2). This increases flexibility in usage, as these dilemmas can be used either in settings that require the traditional action–utilitarian pairing or in settings where the role of action compared with inaction in moral judgments is examined.

Making dilemmas more realistic involves adding information about the people involved and their relations to each other. From a purely cognitive perspective, one might deem this information unnecessary. However, for external validity, we argue that adding details and humanizing the protagonists (e.g., by mentioning their gender, nationality, or relationship to oneself) is useful (Hester & Gray, 2020). If a manipulation only influences dilemma judgments when the situation descriptions are devoid of any personal or social information, the generalizability of its influence is limited. On the downside, however, the richer descriptions present in factual dilemmas likely decrease effect sizes because the additional information introduces unsystematic variance. Thus, we recommend increasing the sample size when using factual dilemmas.

Factual dilemmas vary along some dimensions that have previously been found to influence dilemma judgments (e.g., Christensen et al., 2014; Moore et al., 2008), for example, whether the protagonist would also benefit from the suggested action or is a disinterested bystander (see Tables 1 and 2 for each dilemma’s level). These variations could be either seen as potential confounds or as desirable to ensure the robustness of any examined effect (note that the present results are very similar when controlling for these factors, see Supplemental Materials). When regarded as confounds, dilemmas only from one combination of these categories (dilemmas where the victim is only going to suffer in one of the options and where no option involves self-benefit) could be selected. Alternatively, to examine generalizability, these dilemma dimensions could be systematically examined, statistically controlled, or allowed to vary.

## Conclusion

Despite widespread criticism, trolley-type dilemmas are used in the overwhelming majority of studies on moral dilemma judgments. By the use of trolley-type dilemmas, evidence for the influence of various psychological processes on dilemma judgments has been accumulated. We suggest that it is time for using more varied and more realistic stimuli. Using more varied dilemmas in psychological studies would inform us how specific or general previously examined cognitive processes are in their influence on judgments, showing how much morality theories generalize. Using more realistic dilemmas should increase participant engagement, leading to more valid results. Here, we suggested new dilemmas based on facts. We showed that factual dilemmas are more realistic than trolley-type dilemmas and induce higher participant engagement. From this, we tentatively conclude that factual dilemmas are suitable stimuli for moral psychology research.

## Supplemental Material

sj-docx-1-psp-10.1177_01461672221103058 – Supplemental material for Deontology and Utilitarianism in Real Life: A Set of Moral Dilemmas Based on Historic Events Click here for additional data file. Supplemental material, sj-docx-1-psp-10.1177_01461672221103058 for Deontology and Utilitarianism in Real Life: A Set of Moral Dilemmas Based on Historic Events by Anita Körner and Roland Deutsch in Personality and Social Psychology Bulletin

sj-docx-2-psp-10.1177_01461672221103058 – Supplemental material for Deontology and Utilitarianism in Real Life: A Set of Moral Dilemmas Based on Historic Events Click here for additional data file. Supplemental material, sj-docx-2-psp-10.1177_01461672221103058 for Deontology and Utilitarianism in Real Life: A Set of Moral Dilemmas Based on Historic Events by Anita Körner and Roland Deutsch in Personality and Social Psychology Bulletin

## Notes

1. We took no measures to exclude participants from taking part in more than one data collection wave, an oversight that was remedied in Study 2.

2. We deleted data from six additional participants who started but (for logistic reasons) did not complete the study.

3. The filler scenarios differed from the dilemmas in that there was no clear contrast between deontology (norm congruence) and utilitarianism (outcome maximization). These scenarios frequently contrasted a self-serving option with a prosocial option.

4. Adding covariates that have previously been found to influence dilemma judgments (see Tables 1 & 2) leads to qualitatively identical results, see Supplemental Materials.

5. We deleted data from 47 additional participants who started but did not complete the study and 2 participants who started the study more than once.

6. This time, we did not reiterate the outcome of the mentioned action.

7. Here, we did not tell participants which dilemmas were based on facts. However, in future research, labeling factual dilemmas as actually based on true facts should increase participants’ experience studies as meaningful, further improving motivation and engagement (see Valsesia et al., 2017).
