# Supplementary material for: Deontology and Utilitarianism in Real Life: A Set of Moral Dilemmas Based on Historic Events
Source: Pers Soc Psychol Bull. 2022 Jun 24;49(10):1511–28. doi: 10.1177/01461672221103058 (PMC10478346; doi:10.1177/01461672221103058)
Supplement: sj-docx-1-psp-10.1177_01461672221103058 – Supplemental material for Deontology and Utilitarianism in Real Life: A Set of Moral Dilemmas Based on Historic Events [file sj-docx-1-psp-10.1177_01461672221103058.docx]

**Factual Dilemmas from Körner & Deutsch: Dilemmas from Real Life: Increased Realism Evaluations for Dilemmas Contrasting Deontology and Utilitarianism Based on Historic Events**

*For some dilemmas, there are two versions, the original version used in the paper, and an improved version modified after data collection, which therefore is not tested yet. These modifications are meant to strengthen the moral implications and prevent misunderstandings.*

**English Versions**

**Factual–Killing (action coincides with utilitarian option)**

1. Conjoined Twins

A few weeks ago, you and your partner became parents of a pair of conjoined twins. The two girls share some organs; for example, they have only one bladder between them. The weaker infant has reduced brain and heart activity and very little functioning lung tissue; whereas, in the stronger infant, all non-shared organs are normally developed. The physicians tell you that currently the stronger twin’s organs keep the weaker one alive. However, after a few months’ growth, the organs will not be able to handle this double strain anymore. The physicians suggest separating the two girls and giving the shared organs to the stronger girl.

If you decide against the operation, according to the physicians, both girls will die soon. If you decide in favor of the operation, the weaker infant will die during the operation, but the stronger one has a very good chance to survive and might have a largely unimpaired life.

How appropriate is it for you to let your twins be separated?

1. Rope ladder

It is 1987 and you are on a ferry from Belgium to England. Suddenly, the ferry starts tilting and water begins to pour in. You and many other passengers are trying to get to the deck by a rope ladder. You are currently halfway up the ladder. Directly above you, a man who seems frozen into immobility by fear or cold is blocking the ladder. You try to speak to him, but he does not react. People behind you are jostling. The ship seems to be sinking fast and the man above you is still blocking the ladder. From the crowd below, someone shouts that you should push the man off.

If you push the man off the ladder, he will probably die, but you and the other people will be able to climb on deck. If you do not push the man off the ladder, he will probably continue blocking the way so that many of the people behind you will not be able to get on deck and therefore will drown.

How appropriate is it for you to push the man off the ladder?

1. Cannibalism (on Lifeboat)

It is 1884 and you are one of four crew members on a small yacht which you are transferring to Australia. In a severe storm in the Indian Ocean, the yacht sinks. The four of you are able to get into its lifeboat and take navigation instruments and food with you. After a few weeks in the lifeboat, your food and water have been gone for some time and there still is no help. The youngest person, a 17-year old cabin boy, has been so thirsty as to drink sea water, and consequently is weakened and in pain. He hardly reacts anymore when you speak to him. The captain suggests killing the boy and feeding on his body.

If you do not kill the boy, all of you will die in the next few days. If you kill the boy, the rest of you will survive for a few weeks longer and could be rescued during this time.

How appropriate is it for you to kill the boy?

1. Doodle bugs

You are the British prime minister during World War II when the Germans begin to use long-distance missiles and hit London. Most missiles do not hit the center but instead hit southern quarters where no important military installations are located. Instead, it is a mostly residential district populated by poorer classes. The population density is comparatively low, leading to fewer casualties than if the bombs did hit their mark. The Germans know roughly but not exactly where the bombs are hitting. By spreading wrong information through spies, you could make them believe that their bombs hit rather the north of the center. As a result, they would not improve their aim, leading to most bombs’ reaching even more southern quarters.

If you do not spread wrong information, the Germans will probably improve their aim of hitting the city center and hit vital military installations [and kill many more people]. If you spread wrong information, the bombs will probably continue killing many poorer people in the south of London.

How appropriate is it for you to spread wrong information?

*Improved version of doodle bugs (not empirically tested yet)*

You are the British prime minister during World War II when the Germans begin to use long-distance missiles and hit London. Most missiles do not hit the center but instead hit southern quarters where no important military installations are located. Instead, it is a mostly residential district populated by poorer classes. The population density is comparatively low, leading to fewer casualties than if the bombs did hit their mark. The Germans know roughly but not exactly where the bombs are hitting. By spreading wrong information through spies, you could make them believe that their bombs hit rather the north of the center. As a result, they would not improve their aim, leading to most bombs’ reaching even more southern quarters. Thus, spreading wrong information will lead to fewer people dying and will also protect military institutions.

If you do not spread wrong information, the Germans will probably improve their aim of hitting the city center and hit vital military installations and kill many more people. If you spread wrong information, you make the enemy target your poorer population in the south of London.

How appropriate is it for you to spread wrong information?

1. Plague spread

In 1665 you are the parish priest in a village with 800 inhabitants in England when there is an outbreak of the plague. After the first deaths, the inhabitants ask you to take charge and make all important decisions for the village. You know that the plague spreads by contagion. Therefore, you consider isolating your village to prevent a spread to nearby villages. However, this will lead to closer contact within the village, increasing the risk of contagion for the people in your charge - among them many children.

If you isolate your village, the probability of a general spread of the plague is reduced. However, the number of deaths in your village will increase. If you do not isolate your village, people will be able to try to save themselves and their families. However, this will likely carry the plague elsewhere, leading to the deaths of many more people.

How appropriate is it for you to isolate your village?

1. Euthanasia (Ghetto)

It is 1942, you are a physician in a hospital of the Jewish Ghetto in Warsaw and are responsible for the infants’ and children’s ward. The Nazis, who are occupying Warsaw, begin to clear the hospital and deport all patients. You feel sure that all children in your unit will be deported and eventually killed because they cannot be used for hard labor. You consider giving the children a lethal dose of morphine before bedtime in order to save them from deportation.

If you kill the children with an overdose of morphine, you will prevent their probable suffering. If you do not kill the children, you will leave them at the mercy of the Nazis.

How appropriate is it for you to give morphine to the children and infants?

1. Immunization (smallpox)

You are a physician in England in 1796 when one of your colleagues discovers that an infection with the harmless cowpox might have made his patients immune to the dangerous smallpox. Smallpox are highly contagious and often deadly. If an infection with cowpox really leads to immunity against smallpox, many lives could be saved by infecting people with cowpox. You consider testing this by infecting a patient first with cowpox and then with smallpox. However, by doing so, you would endanger this person’s life. You cannot test the procedure on yourself because you have already survived smallpox and are immune. You search for volunteers, but the only offer is from your gardener who offers his eight-year-old son.

If you infect the boy with both diseases, you will risk his life but will be able to test your immunization procedure, which might save many lives. If you do not infect the boy, you will not endanger him, but you cannot test the procedure, and people will continue to die of smallpox.

How appropriate is it for you to infect the boy with cowpox and smallpox?

1. 911

You are passenger on a flight from New Jersey to San Francisco when four terrorists take over the plane and claim to have a bomb on board. Some passengers are able to phone friends or family and discover that two other planes have been hijacked in New York and crashed into the World Trade Center. You think that the terrorists on board also plan to crash this plane into a building.

If you do not resist the hijackers, they might crash the plane into another building, which would kill all the passengers as well as many other people. If you resist the hijackers, they will probably start killing passengers or detonate their bomb. However, you could cause the plane to crash in an uninhabited area which would still kill all passengers but would prevent the death of additional people.

How appropriate is it for you to resist the terrorists?

1. Coma (mother)

Since a riding accident several years ago, your mother is in a persistent vegetative state. Her brain is permanently damaged. She still breathes on her own and has reflexes. During the day, her eyes are open and moving, but she cannot consciously perceive anything. She has no higher brain functions left and there is no hope of improvement. However, she could continue living like this for a long time. You and your relatives are suffering immensely because of her condition. In your opinion, the situation robs her of all dignity. Now, after 7 years, you consider suffocating her with a towel.

If you kill your mother by suffocation, you will release yourself and your relatives from suffering. If you do not kill her, your mother will continue living in this state, probably for many years.

How appropriate is it for you to suffocate your mother?

*Improved version of Coma (not empirically tested yet); dignity removed*

Since a riding accident several years ago, your mother is in a persistent vegetative state. Her brain is permanently damaged. She still breathes on her own and has reflexes. During the day, her eyes are open and moving, but she cannot consciously perceive anything. She has no higher brain functions left and there is no hope of improvement. However, she could continue living like this for a long time. You and your relatives are suffering immensely because of her condition. Now, after 7 years, you consider suffocating her with a towel.

If you kill your mother by suffocation, you will release yourself and your relatives from suffering. If you do not kill her, your mother will continue living in this state, probably for many years.

How appropriate is it for you to suffocate your mother?

1. Organ donation law

You are director in the French Agency for Biomedicine. In France, all people are organ donors unless they opt out. Still, many people die every year because they do not receive a donor organ in time. You are responsible for deciding whether a new system that uses a changed death criterion should be tested in nine hospitals. Until now, organs are only allowed to be harvested when the patient’s brain activity has completely stopped. However, by that time, organs often cannot be transplanted anymore and become useless. The new criterion would allow organs to be harvested at an earlier time. Specifically, organs could be taken if patients have cardiac arrest that persists after attempts at resuscitation. This procedure would result in a large increase of transplantable donor organs. However, in rare cases, patients have been known to survive after they met this criterion. These people would then die because of the organ removal.

If you authorize the pilot phase of the new criterion, you will risk killing people by organ harvesting who might otherwise have survived. However, the new criterion would save many more lives by enabling a much larger number of organ transplants. If you veto the new criterion, many people will die every year because they will not get a donor organ.

How appropriate is it for you to introduce the new organ donation criterion?

1. Still births

It is 1944 and you and four other inmates are working as nurses in the women’s hospital ward of the Auschwitz-Birkenau concentration camp. One of your duties is to help pregnant inmates when they give birth. However, you know that the SS guards have the order to kill mother and baby if the baby is born alive. In the case of stillbirths, on the other hand, the mothers are usually not killed. Consequently, you consider killing the babies by injecting poison shortly before birth to at least save the mothers.

If you kill the babies before birth, the mothers will most likely be saved. If you help deliver the babies alive, both, babies and mothers, will be killed by the SS guards.

How appropriate is it for you to poison the babies?

1. Tyrannicide

You are an army officer in the Dominican Republic in 1961. For decades now, your country is governed by the dictator Rafael Trujillo who has had thousands of people killed because of their ethnicity or their political views. You are a member of a group that wants a new start for your country without a civil war. To achieve this goal, you consider it unavoidable to kill the dictator in order to disempower him, his family, and his followers. For this, you and six co-conspirators are asked to ambush and kill the dictator.

If you kill the dictator, your country will have a chance for a new start. If you do not kill him, the regime will continue its killings.

How appropriate is it for you to kill the dictator?

1. Real lifeboat

It is 1841 and you are a crew member on a ship. After colliding with an iceberg, the ship sank. You, some other crew members, and passengers were able to save yourselves onto two lifeboats. The two boats took off in different directions to increase the chances of being found. The next day, it starts to rain. Your lifeboat is overcrowded with 41 people. You navigate while the others scoop out the water and oar. Nevertheless, the large waves could make your boat sink at any time. You think that approximately ten adults would have to leave the boat to substantially increase the chance that the other passengers will survive. However, the water is so cold, that they would freeze to death.

If you do not throw anybody over board, the boat will most likely sink within the next few hours and all 41 people will die. If you instead throw ten people over board, the rest of the passengers will be able to keep rowing towards land and will have a good chance of being rescued.

How appropriate is it for you to throw ten people over board?

1. Septuplets

You and your partner are a couple in the USA when you learn that you are pregnant with septuplets (7 babies). The risk that all or some of the children will be born with severe disabilities or not survive at all is extremely high. Therefore, the doctors recommend aborting some of the fetuses in order to increase the chances of survival for the others; their recommendation is to abort 3 fetuses. The remaining fetuses would have a much higher chance of being born healthy.

If you abort three fetuses, you will increase the chance of survival and health for the remaining four fetuses. If you do not abort any of the fetuses, there is a high risk that all or most of them will die or have severe disabilities.

How appropriate is it for you to abort three of the fetuses?

1. Assisted suicide state

You are a member of the Canadian Senate and have to vote on a new law. Canada’s health care expenses are increasing steadily, mainly because the ratio of old people increases. Recent studies indicate that the legalization of medically assisted suicide would strongly decrease medical expenses. The state considers legalizing medically assisted suicide for the patients who are terminally ill and whose life expectancy is 6 months or less. If they wish, these patients could then choose to be killed by a doctor with an overdose of tranquillizers. Experts estimate that the new law would save 139 million dollars. This money could be used for other medical treatments.

If you allow medically assisted suicide, many more people will get better medical treatment improving their quality of life and life expectancy. However, you would legalize a form of killing. If you do not allow medically assisted suicide, the health care system will have to keep on saving money in every possible way.

How appropriate is it for you to change the law and make medically assisted suicide legal?

1. Atom bomb

You are a physicist researching nuclear energy and have been living in the USA for some years. In 1939, there are rumors that the Nazi regime is building the first atomic bomb. You assume that Germany would not dare to use its bomb if it was known that the US had atomic bombs, too. Therefore, you consider asking the US president to command the construction of the atomic bomb in America and to promise to contribute to its development.

If you initiate the construction of an American atomic bomb, you could prevent the Germans from using their bomb. At the same time, you would take part in the construction of the deadliest weapon of mass destruction in history.

How appropriate is it for you to initiate the construction of the atomic bomb?

**Factual–Saving (action coincides with deontological option)**

1. Medicine costs

You are the Belgian minister of health. A 7-year-old boy who suffers from a very rare immunological disease asks you for a refund for his medicine. Without taking this medicine regularly, the boy will die. Currently, the medicine is not covered by the health care system, and it is so expensive that the family, who has been buying the medicine for years, will not be able to raise enough money for much longer (approximately 200,000 euros per year). Thus, the boy will die, if you refuse the refund.

If you refuse to pay for the medicine, the boy will probably die within the next few years. However, if you pay, this money will have to be saved elsewhere; thus, other treatments will not be able to be covered. Funding the boy will therefore likely lead to the death of several other people.

How appropriate is it for you to fund the boy’s medicine?

1. RAF

You are the German head of state in 1977 and the RAF, a far-left terroristic group has kidnapped the President of the Federation of the German Employers’ Association. In exchange for his life, the RAF demands the release of eleven imprisoned RAF-members. Two years ago, the RAF has successfully obtained the release of five RAF-members by another kidnapping. Four of the released members have since then committed further terrorist attacks where people were killed.

If you consent to the RAF’s demands, eleven former terrorists will be released and will likely commit further terrorist attacks in which many people could get killed. If you refuse, the hostage will be killed.

How appropriate is it for you to release the former terrorists?

1. Mare nostrum *(Note: depending on what the refugees’ future would be when not fleeing, one might come to a different conclusion what the utilitarian option is from the stated one)*

You are a member of the British Ministry of Foreign Affairs. Many refugees from Northern Africa try to cross the Mediterranean Sea because of war or lack of economic perspectives. The EU runs a rescue program, “Mare Nostrum”, that has already saved 150,000 refugees from drowning. Illegal human trafficking gangs abuse this program. For huge sums of money, they give non-seaworthy boats to refugees and point out that they will be rescued if they get into difficulties. However, this year 3,000 refugees have drowned or died of thirst because they were not rescued in time. Your government thinks about stopping the rescue program in order to end human trafficking and scare off refugees. At first, the number of people dying at sea would increase, but in the future, probably fewer people would try to cross the sea, leading to fewer deaths in the long run.

If you continue the program, you could save lives now, but in the long run, thousands of refugees will continue to die at sea. If you stop the program, refugees and human trafficking will be discouraged, but refugees will be left to die at sea.

How appropriate is it for you to continue the program to save refugees in the Mediterranean?

1. Rwanda

You are a project leader in a development aid team in Rwanda. All over the country, members of the ethnic group of Hutu attack members of the Tutsi group. The family of one of your Tutsi employees is hiding in your agency’s center. Another employee arrives and tells you that he just witnessed his whole family and another of your employees being killed by an armed group simply for being Tutsis. Shortly afterwards, a crowd of armed people gathers at the center. The leader threatens to attack the building and kill everybody inside unless you hand over your employee who just arrived.

If you protect your employee, the mob will probably attack the center and kill many people. If you hand over your employee, he will be killed but the other people under your care will be safe.

How appropriate is it for you to protect your employee?

*Improved version of Rwanda (not empirically tested yet); make more clear that protecting is the action*

You are a project leader in a development aid team in Rwanda. All over the country, members of the ethnic group of Hutu attack members of the Tutsi group. The family of one of your Tutsi employees is hiding in your agency’s center. A crowd of armed people gathers at the center. Another employee arrives and tells you that he just witnessed his whole family and another of your employees being killed by an armed group simply for being Tutsis. The leader threatens to attack the building and kill everybody inside if you receive the employee who just arrived in your center and protect him.

If you protect your employee, the mob will probably attack the center and kill many people. If you do not protect your employee, he will be killed but the other people under your care will be safe.

1. Sahara ransom

You are the German head of state and 10 of your citizens are taken hostage in the Sahara Desert by a group of terrorists. You have to decide whether to pay a ransom of several million euros. Other measures to free the hostages have been tried but did not succeed; by now, it has been going on for half a year and one woman has died. Similar situations in the past have shown that terrorists use the money to buy weapons and kill even more people. Furthermore, future tourists will more likely become targets if you pay.

If you pay the ransom, you can save the hostages. If you do not pay, the hostages will be killed, but you will prevent future terrorism being financed, which will probably save more lives.

How appropriate is it for you to pay the ransom?

1. Aid for Syria

There is a Civil War between the Syrian regime and rebels that has been going on for years. By now, the humanitarian situation is horrible – the people have neither sufficient food nor adequate medical care. You are an employee for the UN and are responsible for the payment of aid funds. Tens of millions of dollars have been approved to go to local Syrian organizations. However, all local organizations are controlled by the Syrian regime. The regime is strongly suspected to only give aid to their own territories and even to misappropriate money to finance the war. Therefore, many other organizations have stopped giving monetary aid to Syria so as not to support the brutal Syrian regime. Nevertheless, the payments could help many suffering people and save lives.

If you hand out the payments, you will help many people but would also unintentionally support the Syrian regime. If you refuse the payments, millions of people will not receive aid anymore.

How appropriate is it for you to give the funds to Syrian organizations?

**Factual Other proscriptive norms (action coincides with utilitarian option)**

1. Rescue torture

You are the chief of police investigating the kidnapping of a banker’s son. You have arrested a suspect who picked up the ransom money. The suspect has already admitted kidnapping the boy but refuses to reveal the hide-out. The suspect has been under arrest for more than 2 days now and you are worried that the kidnapped boy may die of thirst soon. You consider using threats or physical violence to make the suspect reveal the hide-out.

If you break the law by torturing the suspect, he will most likely reveal the hide-out. If you adhere to the law by not torturing the suspect, he might remain silent about the hideout for another few days, which could lead to the boy’s death.

How appropriate is it for you to torture the suspect?

1. War torture

It is 1940 and you are the head of a British institution that interrogates German prisoners of war. Some of the inmates might have important war-related information that you could use to save thousands of lives. However, most prisoners probably have no relevant information. In the current war phase, there is a lot at stake for Great Britain. France is being heavily invaded by the Germans and the German air force has started to bomb British cities. Therefore, your superiors demand quick results – by any means. You think about systematically torturing all inmates to get war-related information. This would clearly break the Geneva Convention which requires Great Britain to protect all people who are no longer fighting, including prisoners of war.

If you decide to systematically torture all inmates, you will probably save thousands of human lives. If you do not torture inmates, you might lose crucial life-saving information.

How appropriate is it for you to torture the inmates?

1. Rugby cannibalism

You are member of a rugby team flying from Montevideo to Santiago de Chile in 1972. The plane crashes, killing 15 of the 40 people on board. You are in an inaccessible region and have no means of communication. However, you do have a radio. You are now at an altitude of 13,000 feet/4,000 meters at -20°F/-30°C. After ten days, only 19 of you are still alive, you have no food left and you hear on the radio that the search for your plane has been given up. You and the others are contemplating whether to eat the remains of your dead team mates, violating their dignity and peace of the dead.

If you and the rest of the survivors eat human bodies, you will survive longer and increase your chances of being rescued. If you and the others do not eat dead bodies, you will starve to death soon.

How appropriate is it for you to eat the remains of your dead team mates?

1. Robin Hood

You are an employee of a bank in Germany. You have many loyal customers who trust in you and your bank. Among them are some customers who are in debt and do not fulfill conditions for being granted a loan. Interviews with them are very stressful for you and for the costumers. Currently, there is one very severe case where a customer and his family would be ruined without a loan. You see an undetectable way to temporarily redirect some deposits and investments from other customers to his bank account which would improve his credit rating sufficiently. This way you could help him as well as other customers in difficulties. At the same time, you would abuse the trust of the wealthy customers and of your superiors and commit fraud.

If you temporarily redirect a part of the wealthy customers’ money, you will be able to help indebted costumers but abuse trust. If you follow the rules, your indebted customers and their families will go bankrupt.

How appropriate is it for you to redirect money from wealthy customers?

1. Knee operations

You are a physician in a hospital in a town in Germany. Your hospital needs to perform at least 50 knee surgeries per year in order to have them refunded by health insurance. Until this December, the hospital has only conducted 48 surgeries; thus, maybe none of the surgeries will be covered by insurance. The clinic has already been in debt the previous year and will have to file for bankruptcy if there will be another debt when the knee surgeries are not refunded. Without this hospital, the region will not have access to fast intensive emergency care. Sooner or later, having no fast access to intensive emergency care will lead to avoidable deaths in the region. The only way of sustaining the clinic would be by performing two knee surgeries that are medically not indicated.

If you perform two unnecessary surgeries, you can save the hospital from bankruptcy and the region will continue to have fast emergency care, but you will perform surgery on two patients for whom other treatments would be more appropriate. If you do not perform the surgeries, the clinic will be closed leading to deaths sooner or later.

How appropriate is it for you to perform the two surgeries that are not medically indicated?

1. Organ market

You are living in Israel and, for the past four years, you have been dependent on dialysis because of kidney failure. No one in your family is a suitable organ donor. The official waiting list is so long that many people die because they never get a donor kidney. After a minor heart attack, you are pushed down further on the waiting list, making it virtually impossible that you will receive a kidney. You think about buying a kidney on the black market where people from poor countries sell their kidneys to wealthy people. The medical risks are low. The donors can live on with one kidney but are medically restricted in what they can do and their health will become more fragile. The black market for organs is well organized but illegal because it pressures poor people in distress to risk their health.

If you do not buy a kidney, you will die in a few years. If you buy a kidney, both you and the donor will probably be able to live well for decades. At the same time, you are abusing the poverty of the donor.

How appropriate is it for you to buy a kidney from the black market?

1. Tax fraud detection

You are the state minister of finance of a German federal state. You have to decide whether or not to buy illegally gained data. You have been offered to buy data about tax evaders. The data were compiled by hackers who have illegally invaded the servers of several foreign banks, where some of your wealthy citizens transfer their money to evade taxes. The data are probably reliable and consists of up to 50,000 transactions.

If you buy the data, you will use illegally obtained information and reward hackers for data theft but will be able to prosecute tax evaders, whose fines will amount to millions. If you do not buy the data, you will miss out on millions of euros of additional tax payments and fines.

How appropriate is it for you to buy the data?

1. Ashley treatment

You are a physician and specialist in medical ethics working in the USA when the parents of a 6-year-old girl consult you. Their daughter suffers from static encephalopathy; she is neither able to walk nor to sit. Developmentally, she will always be at the level of a 3-month-old baby. Her parents are still able to take care of her at home, but the girl is growing. To prevent their daughter from becoming too heavy, the parents want to stop her growth and inhibit her sexual development by removing her uterus and breast tissue and by giving her a high-dose estrogen therapy. The treatment is not medically necessary, and the girl is unable to decide for herself. The treatment would therefore violate the child’s right to physical integrity.

If you do not allow the treatment, the parents will soon be unable to take care of the girl and she will not be able to stay in her family environment. If you allow the treatment, the girl will undergo unnecessary treatment and operations but will be able to stay with her parents.

How appropriate is it for you to perform the surgery and the treatment on the child?

1. Test tube sibling

You are a judge at a British court of appeal and have to decide about a case of test-tube fertilization. A boy suffering from beta-thalassemia, a deadly blood disease, can only be saved by a stem cell or bone marrow donation. However, despite all efforts, no suitable donor could be found. The parents want to save their child by having another baby whose stem cells fit. With the help of test-tube fertilization, a genetically suitable embryo would be selected and then implanted into the uterus. If the pregnancy and, after birth, the transplantation of the stem cells from the umbilical cord are successful, the chances that the sick child recovers are above 90%. However, this would mean breeding a baby as a stem cell donor.

If you allow the genetic selection, the sick child will probably be saved and recover completely. If you do not allow the genetic selection, the sick child will die (unless another donor can be found, which is very unlikely).

How appropriate is it for you to allow genetic selection of a child?

1. Lion hunt

You are working at the nature conservation agency “International Union for Conservation of Nature in Tanzania”. You are responsible for the protection of endangered species, notably lions, whose population is steadily declining in the wild. Research has shown that the increase of agricultural areas is the main reason for this. To provide a long-term habitat for lions, you want to buy land as a conservation area. In spite of your immense efforts to raise money, you are unable to raise the necessary funds. You consider killing a few of the threatened lions and selling their furs for 60,000 dollars apiece, which would be enough money – much more than any other action you have tried or can think of.

If you kill some of the lions, you will have enough money to buy land for the protection of the remaining lions. If you do not kill any lions, you will not have the funds to buy an adequate size of land.

How appropriate is it for you to kill some of the lions?

**Factual Other prescriptive norms (action coincides with deontological option)**

1. Bishop

You are a catholic archbishop in the Netherlands when the German occupiers begin to deport Dutch Jews in 1942. You are aware that the deported people suffer a lot, even though you do not know any details. You are thinking about publicly censuring the deportations. However, by a silent agreement with the Germans, you could assure that at least Christians with Jewish heritage are being spared. If you speak up, you would risk the deportation of the Christians with Jewish heritage, including members of your own community.

If you remain silent about the deportations, Dutch Jews will continue to be deported, but the Christians with Jewish heritage will remain free. If you publicly censure the deportations, you will risk the life of all Christians with Jewish heritage, but you will fulfill your moral duty to call attention to the wrongful deportations.

How appropriate is it for you to openly censure the deportations?

1. Trail of Tears

You are one of the leaders of the Native American Cherokee in 1830. The US Congress has decided to relocate all southern Native American tribes to the west. So far, you have been opposing relocation because forced relocation violates your rights, which the US constitution guarantees to all US citizens and Native Americans. The Supreme Court has already decided in your favor, but you know that the white settlers and the US President will not accept your refusal to move. War with the US armed forces is inevitable if you do not move, which you have no chance to win.

If you advocate for voluntary relocation, chances will be high that your tribe can move without bloodshed, but you will irrevocably give up your tribe’s traditional territory. If you further advocate against the relocation, there will be war with the US armed forces and many of your fellow tribe members will die.

How appropriate is it for you to continue resisting against relocation?

1. Veterinarian

You are a European veterinarian and do animal experiments on highly contagious germs. On a daily basis, you cause suffering to animals by infecting them with deadly diseases. You are aware that this work provides important insights that, in the long run, can save many other animals. Furthermore, you are a specialist and because of your expertise you can achieve more than other veterinarians. However, you have become a vet in order to heal animals and you are considering quitting your job.

If you quit your job, fewer animals will die in the lab, but there will also be fewer findings about animal diseases, leading to many deaths. If you continue your job of infecting animals with diseases, you will keep causing suffering to animals, but your research will probably save the life of many other animals.

How appropriate is it for you to quit your job?

1. Hogesa

You are a judge at an administrative court in Germany. A right-wing group wants to organize a demonstration against Islamist extremism. Freedom of speech and freedom of assembly are inalienable basic rights. However, at former demonstrations of this group, there have repeatedly been violent attacks against immigrants and the police.

If you prohibit the demonstration, you will deny the group’s freedom of assembly and freedom of speech. If you allow the demonstration, there is a risk that people will be harmed.

How appropriate is it for you to allow the demonstration?

1. Mass rape

You are a judge in Germany in the early 1990s. At a fair, a group of eight drunk men raped a 17-year-old waitress. One of the men did not participate but called the police. When the police arrived, they found the girl injured and unconscious. It is clear which group was responsible, but the investigations were not able to determine who of the group is guilty; you know that at least one of them is innocent, but many others (probably all) committed rape. You have just finished interrogating them. The 8 men keep silent, the girl’s recollections are vague, and the evidence from the girl’s body is unusable. There seems to be no way to find out who is guilty and who is innocent.

If you keep all men in investigation custody, the innocent man will be jailed wrongfully. If you drop the charges, several rapists will be released.

How appropriate is it for you to release all eight men?

*Improved version of Mass rape (not empirically tested yet)*

You are a judge in Germany in the early 1990s. At a fair, a group of eight drunk men raped a 17-year-old waitress. One of the men did not participate but called the police. When the police arrived, they found the girl injured and unconscious. It is clear which group was responsible, but the investigations were not able to determine who of the group is guilty; you know that at least one of them is innocent, but many others (probably all) committed rape. You have just finished interrogating them. The 8 men keep silent, the girl’s recollections are vague, and the evidence from the girl’s body is unusable. There seems to be no way to find out who is guilty and who is innocent.

If you keep all men in investigation custody, the innocent man will be jailed wrongfully. If you drop the charges, several rapists will be released increasing the probability of their committing crimes in the future.

How appropriate is it for you to release all eight men?

1. Endowment

After a long-term imprisonment, a convicted child kidnapper and murderer wants to organize a foundation for young victims of crime. You are the president of the agency who decides about the foundation. The founder would offer a seed capital of 25,000 euros. However, the foundation would be named after the offender and it is unclear whether he is acting out of remorse or simply to clear his name. The family of his victim opposes such a foundation and considers it immoral, derogatory, and an exploitation of the victim.

If you oppose the foundation, the capital will be lost. If you approve the foundation, crime victims can be helped by the starting capital and additional fund raisings.

How appropriate is it for you to oppose the foundation?

**German Versions**

Note, in Study 1, the questions were used in their long form (including the text in brackets). However, we would generally recommend omitting the information in brackets.

**Factual–Killing (proscriptive norm, i.e., action coincides with utilitarian option)**

1. Conjoined Twins

Sie sind vor ein paar Wochen, am 08.08.2000, Eltern siamesischer Zwillinge geworden. Die beiden Mädchen sind am Unterleib zusammengewachsen und haben einige Organe nur gemeinsam, u.a. teilen sie sich eine Blase. Das schwächere Kind hat eingeschränkte Gehirn- und Herzfunktionen und kaum funktionsfähiges Lungengewebe, während beim stärkeren Kind alle nicht-geteilten Organe normal ausgebildet sind. Die Ärzte sagen, dass die Organe des stärkeren Kindes das Schwächere am Leben halten, was aber nur für wenige Monate möglich ist. Die Ärzte schlagen vor, die beiden Mädchen zu trennen und bei der Operation dem stärkeren Kind die gemeinsamen Organe zu geben.

Wenn Sie die Mädchen nicht operieren lassen, werden, laut Aussage der Ärzte, beide Mädchen in Kürze sterben. Wenn Sie die Mädchen operieren lassen, wird das schwächere Kind noch bei der Operation sterben, während das stärkere Kind sehr gute Überlebenschancen und Chancen auf ein normales Leben hat.

Wie angemessen ist es, dass Sie Ihre Kinder trennen lassen (um das Leben des stärkeren Kindes zu retten)?

1. Rope ladder

Sie befinden sich am 06.03.1987 auf einer Fähre von Belgien nach England, als sich das Schiff plötzlich zur Seite neigt und mit Wasser füllt. Sie und viele andere Passagiere versuchen über eine Strickleiter vom Inneren des Schiffes an Deck zu gelangen. Doch direkt über Ihnen befindet sich ein Mann, der vor Furcht oder Kälte erstarrt scheint und auch nicht reagiert als Sie ihn ansprechen. Die Menschenmenge hinter Ihnen drängt, da das Schiff sehr schnell sinkt, aber der Mann vor Ihnen blockiert seit Minuten die Strickleiter. Aus der Menge hören Sie einen Ruf, dass Sie den Mann von der Leiter stoßen sollen.

Wenn Sie den Mann von der Leiter stoßen, wird er aller Wahrscheinlichkeit nach sterben, aber Sie und die vielen anderen können an Deck klettern. Wenn Sie ihn nicht von der Leiter stoßen, wird er wahrscheinlich so lange die Leiter blockieren, dass viele der Menschen hinter Ihnen ertrinken.

Wie angemessen ist es, dass Sie den Mann von der Leiter stoßen (um sich und die anderen zu retten)?

1. Cannibalism (on Lifeboat)

Sie sind 1884 eines von vier Besatzungsmitgliedern auf einer kleinen Yacht, die nach Australien überführt wird. Durch schwere Unwetter sinkt das Schiff im indischen Ozean. Sie können sich jedoch zu viert mit Navigationsgeräten und sehr wenig Nahrung auf das Beiboot retten. Wochen später ist keine Rettung in Sicht und Ihre Nahrung ist längst aufgebraucht. Das jüngste Mitglied, ein 17-jähriger Schiffsjunge, hat aus Durst Meerwasser getrunken, ist deshalb sehr geschwächt und leidet unter starken Schmerzen. Er ist kaum mehr ansprechbar. Der Kapitän schlägt vor, den Jungen zu töten, um sich von seiner Leiche zu ernähren.

Wenn Sie den Jungen nicht töten, werden Sie alle in wenigen Tagen sterben. Wenn Sie den Jungen töten, werden Sie noch mehrere Wochen überleben und in der Zeit vielleicht gerettet werden.

Wie angemessen ist es, dass Sie den Jungen töten (um sich und die anderen von ihm zu ernähren)?

1. Doodle bugs

Sie sind Großbritanniens Premierminister während des 2. Weltkriegs, als die deutschen Gegner 1944 beginnen London mit Langstreckenraketen zu beschießen. Die meisten Raketen treffen nicht das Zentrum, sondern schlagen in südlichen Stadtteilen ein, wo sich keine wichtigen Militäreinrichtungen befinden. Dort wohnen eher ärmere Menschen, aber die Bevölkerungsdichte ist geringer, sodass pro Bombeneinschlag weniger Menschen sterben, als wenn die Bomben das Zentrum treffen würden. Die Deutschen wissen, dass ihre Bomben nicht weit neben dem Ziel eingeschlagen haben können. Für die Feinanpassung brauchen sie aber Informationen darüber, wo genau die Bomben landen. Indem Sie durch Spione Informationen streuen, dass die Bomben leicht nördlich vom Zentrum einschlagen, könnten Sie dafür sorgen, dass die Bomben in Zukunft noch etwas südlicher einschlagen.

Wenn Sie keine falschen Informationen verbreiten, ist davon auszugehen, dass die Deutschen zukünftig wahrscheinlich besser auf das Stadtzentrum zielen und viele Menschen und militärische Einrichtungen treffen. Wenn Sie falsche Informationen verbreiten, werden die Bomben noch weiter südlich einschlagen und viele Menschen in Südlondon töten.

Wie angemessen ist es, dass Sie falsche Informationen verbreiten und damit Südlondon zur Zielscheibe der Langstreckenraketen machen (um damit das Stadtzentrum zu schützen)?

1. Plague spread

Sie sind Priester in Eyam, einem Dorf in Mittelengland, als dort im Jahr 1665 die Pest ausbricht. Nach den ersten Todesfällen bitten die Dorfbewohner Sie, ab jetzt alle wichtigen Entscheidungen für den 800-köpfigen Ort zu treffen. Sie wissen, dass sich die Pest durch Ansteckung ausbreitet. Deshalb überlegen Sie, das Dorf zu isolieren, um den Kontakt mit umliegenden Orten und damit eine weitere Ausbreitung der Pest zu verhindern. Durch den damit viel engeren Kontakt der Dorfbewohner steigt dann allerdings die Gefahr, dass sich Ihre Schutzbefohlenen, darunter viele Kinder, mit der Pest anstecken und sterben.

Wenn Sie das Dorf isolieren, verringert sich die Wahrscheinlichkeit, dass sich die Pest weiter ausbreitet, doch viele Dorfbewohner werden sterben. Wenn Sie das Dorf nicht isolieren, können alle Dorfbewohner versuchen, sich und ihre Familien zu retten, was aber die Ansteckung in anderen Orten und damit eine weitere Ausbreitung der Pest wahrscheinlicher macht, sodass noch viel mehr Menschen sterben werden.

Wie angemessen ist es, dass Sie das Dorf isolieren (und damit das Leben Ihrer Dorfbewohner gefährden, um damit andere Orte vor Ansteckung zu bewahren)?

1. Euthanasia (Ghetto)

Sie sind Ärztin im Krankenhaus des jüdischen Ghettos von Warschau, als die deutschen Besatzer im Jahr 1942 beginnen, das Krankenhaus zu räumen und alle Patienten zu deportieren. Sie sind für die Kinder- und Säuglingsstation verantwortlich und sind sich sicher, dass alle Kinder deportiert und getötet werden, da die Deutschen die Kinder nicht als Zwangsarbeiter einsetzen können. Sie überlegen daher, den Ihnen anvertrauten Kindern und Säuglingen vor dem Schlafengehen eine tödliche Dosis Morphium zu spritzen, um ihnen so die Deportation zu ersparen.

Wenn Sie die Kinder und Säugling durch eine Überdosis Morphium töten, verhindern Sie, dass die Deutschen den Kindern Leid zufügen und diese ermorden. Wenn Sie die Kinder nicht töten, überlassen Sie diese der Willkür der Besatzer.

Wie angemessen ist es, dass Sie den Kindern und Säuglingen Morphium verabreichen (um diesen die Deportation zu ersparen)?

1. Immunization (smallpox)

Sie sind Arzt in Südengland, als Sie im Jahr 1796 von einem Kollegen erfahren, dass eine Infektion mit den harmlosen Kuhpocken einige seiner Patienten möglicherweise gegen die gefährlichen Pocken immunisiert hat. Die Pocken sind hochansteckend und häufig tödlich. Wenn die Kuhpocken tatsächlich immun machen, könnte man damit viele Leben retten. Sie überlegen, dieses Verfahren zu testen, indem Sie einen Patienten zuerst mit Kuhpocken und dann mit Pocken anstecken. Allerdings gefährden Sie durch die Infektion das Leben des Patienten. An sich selbst können Sie dieses Verfahren nicht testen, weil Sie die Pocken schon überlebt haben und deshalb bereits immun sind. Als Sie nach Freiwilligen suchen, stellt nur Ihr Gärtner seinen achtjährigen Sohn zur Verfügung.

Wenn Sie den Jungen mit beiden Krankheiten infizieren, riskieren Sie sein Leben, können jedoch das neue Immunisierungsverfahren testen, das viele Leben retten könnte. Wenn Sie den Jungen nicht infizieren, dann bringen Sie den Jungen nicht in Gefahr, doch können das Immunisierungsverfahren nicht testen.

Wie angemessen ist es, dass Sie den Jungen mit Kuhpocken und Pocken infizieren (um herauszufinden, ob eine Kuhpockeninfektion gegen Pocken immunisiert und so möglicherweise viele Leben retten kann)?

1. 911

Sie sind im Jahr 2001 Flugzeugpassagier von New Jersey nach San Francisco. Vier Terroristen übernehmen die Kontrolle über das Flugzeug und behaupten eine Bombe an Bord zu haben. Einige Passagiere können per Handy Bekannte erreichen und erfahren, dass in New York zwei Flugzeuge entführt und in das World Trade Center gestürzt wurden. Sie vermuten daher, dass auch Ihre Entführer beabsichtigen, das Flugzeug in ein Gebäude zu stürzen.

Wenn Sie sich nicht gegen die Entführer zur Wehr setzen, besteht die Möglichkeit, dass die Terroristen das Flugzeug in ein weiteres Gebäude stürzen wodurch nicht nur alle Flugzeuginsassen, sondern auch viele andere Menschen sterben würden. Wenn Sie sich gegen die Entführer wehren, werden die Entführer Insassen des Flugzeugs zu töten. Sie würden durch Ihren Widerstand also den Tod Unschuldiger provozieren. Sie können dann aber einen Absturz in einem unbewohnten Gebiet herbeiführen*, wodurch zwar alle Flugzeuginsassen sterben, aber wahrscheinlich sonst niemand*.

Wie angemessen ist es für Sie, sich gegen die Geiselnahme zu wehren (um damit zu verhindern, dass die Terroristen das Flugzeug in ein Gebäude stürzen, auch wenn dann Ihre Mitinsassen getötet werden)?

1. Coma (mother)

Es ist 2012. Ihre Mutter liegt seit einem Reitunfall im Wachkoma. Sie hat keine höheren Gehirnfunktionen mehr, ihr Gehirn ist dauerhaft geschädigt. Trotzdem kann sie selbstständig atmen und hat Reflexe. Sie öffnet tagsüber die Augen und zeigt Augenbewegungen, nimmt jedoch nichts bewusst wahr. Es gibt keine Aussicht auf Besserung, jedoch kann sie in diesem Zustand vermutlich noch lange weiterleben. Sie und ihre Angehörigen leiden sehr stark unter dem Zustand Ihrer Mutter. [Sie empfinden die Lage Ihrer Mutter im Pflegeheim als würdelos.] Nach insgesamt 7 Jahren überlegen Sie Ihre Mutter zu töten, indem Sie sie mit einem Handtuch ersticken, um sie nicht länger leiden zu lassen.

Wenn Sie Ihre Mutter durch Ersticken töten, erlösen Sie sich selbst und alle Angehörigen. Wenn Sie sie nicht töten, bleibt Ihre Mutter auf unbestimmte Zeit im Wachkoma und Sie und die Angehörigen müssen weiter mitleiden.

Wie angemessen ist es für Sie, Ihre Mutter zu ersticken (damit Sie und die anderen Angehörigen nicht mehr leiden müssen)?

1. Organ donation law

Sie sind 2007 Vorstand in der französischen Agentur für Biomedizin. In Frankreich gilt die Widerspruchsregelung, nach der jeder Bürger Organspender ist, sofern er nicht widerspricht. Trotzdem sterben Kranke wegen fehlender Spenderorgane. Sie sollen entscheiden, ob ein Testlauf in neun Kliniken mit neuen Regelungen zur Organspende durchgeführt werden soll. Bis jetzt gilt als Todeskriterium der Hirntod: Es dürfen erst Organe entnommen werden, wenn das Gehirn keinerlei Aktivität mehr zeigt. Die neue Regelung für den Testlauf würde den Herzstillstand als Kriterium festlegen. Dann dürfen Organe entnommen werden, wenn ein Mensch nach Wiederbelebungsversuchen immer noch einen Herzstillstand hat. Allerdings können Menschen mit dieser Art Herzstillstand in seltenen Fällen doch überleben, würden aber durch die Organentnahme getötet. Da der Herzstillstand in der Regel früher eintritt als der Hirntod, würde die neue Regelung die Anzahl der transplantierbaren Organe stark vergrößern.

Wenn Sie den Testlauf mit dem früheren Todeskriterium starten, riskieren Sie, dass Menschen, die eventuell durch weitere Maßnahmen gerettet werden könnten, durch die Organentnahme sterben; jedoch würden, wegen der vielen neuen Spenderorgane, insgesamt mehr Menschen überleben. Wenn Sie bei der alten Regelung bleiben, sterben weiterhin viele kranke Menschen, weil es zu wenige Spenderorgane gibt.

Wie angemessen ist es für Sie, das Todeskriterium zu ändern (und damit mehr wartende Menschen zu retten)?

1. Still births

Sie und vier andere Insassinnen arbeiten 1944 als Arzthelferinnen in der Frauen-Krankenstation des Konzentrationslagers Auschwitz-Birkenau. Eine Ihrer Aufgaben ist es, schwangeren Insassinnen zu helfen, ihre Kinder zur Welt zu bringen. Sie wissen allerdings, dass die SS-Wachleute bei Neugeburten klare Anweisungen haben, wie mit Mutter und Kind zu verfahren ist: Wenn das Kind lebend zur Welt kommt, werden Mutter und Kind ermordet. Bei Totgeburten hingegen werden die Mütter meistens verschont. Sie überlegen daher, die Kinder kurz vor der Geburt durch eine Injektion von Gift zu töten, um so wenigstens die Mütter zu retten.

Wenn Sie alle Kinder kurz vor der Geburt töten, werden die Mütter sehr wahrscheinlich verschont. Wenn Sie die Kinder lebend zur Welt bringen, werden Mütter und Kinder von den SS-Wachleuten getötet.

Wie angemessen ist es für Sie, die Kinder zu töten (um die Mütter zu retten)?

1. Tyrannicide

Sie sind 1961 Offizier in der Armee der Dominikanischen Republik. Seit Jahrzehnten wird Ihr Land vom Diktator Rafael Trujillo regiert, der schon tausende Menschen aufgrund ethnischer Zugehörigkeit oder politischer Orientierung ermorden ließ. Sie sind Teil einer Gruppe, die einen Neuanfang für das Land ohne Bürgerkrieg möchte. Hierzu ist es Ihrer Einschätzung nach unausweichlich, den Diktator zu ermorden, um so ihn selbst, seine Familie und seine Anhänger zu entmachten. Sie und sechs Mitverschwörer sollen daher den Diktator in einem Hinterhalt töten.

Wenn Sie den Diktator töten, hat die Dominikanische Republik eine Chance auf einen Neuanfang. Wenn Sie den Diktator verschonen, werden weiterhin tausende Menschen dem blutigen Regime zum Opfer fallen.

Wie angemessen ist es für Sie, den Diktator zu töten (um viele andere Menschenleben zu retten)?

1. Real lifeboat

Sie sind 1841 Crewmitglied auf einem Schiff, das nach Kollision mit einem Eisberg sinkt. Sie, weitere Crewmitglieder und Passagiere konnten sich auf zwei Rettungsboote retten. Die beiden Boote trennen sich, um leichter gefunden zu werden. Am nächsten Tag setzt Regen ein und Ihr Rettungsboot, das mit 41 Personen ohnehin überbelegt ist, schlägt leck. Sie navigieren, während die anderen ununterbrochen Wasser abschöpfen und rudern. Trotzdem füllt sich das Boot mit Wasser. Wegen der hohen Wellen könnten Sie jederzeit untergehen. Sie denken, dass ca. 10 Erwachsene das Boot verlassen müssten, um die Überlebenschancen der anderen deutlich zu erhöhen. Das Wasser ist aber so kalt, dass diese nach kurzer Zeit erfrieren würden.

Wenn Sie niemanden über Bord werfen, werden Sie aller Wahrscheinlichkeit nach in den nächsten Stunden untergehen, sodass alle 41 Personen sterben. Wenn Sie 10 Personen über Bord werfen, werden Sie noch mehrere Tage in Richtung Land rudern können, womit Sie und die restlichen Überlebenden gute Chancen hätten, gerettet zu werden.

Wie angemessen ist es, dass Sie 10 Personen über Bord werfen (um damit die Überlebenschancen für den Rest deutlich zu erhöhen)?

1. Septuplets

Sie sind im Jahr 2014 ein Ehepaar in den USA und erfahren, dass Sie Siebenlinge erwarten. Das Risiko, dass die Kinder mit schweren Schädigungen zur Welt kommen oder gar nicht lebensfähig sind, ist extrem hoch. Der Arzt empfiehlt daher, Föten zu töten um die Überlebenschancen der verbleibenden Föten zu erhöhen. Damit hätten die verbleibenden Föten eine höhere Chance gesund geboren zu werden. Der Gynäkologe rät zur Abtreibung von drei Föten.

Wenn Sie die Abtreibung der drei Föten vornehmen, erhöhen Sie die Chancen, dass wenigstens vier der Föten überleben und gesund zur Welt kommen. Wenn Sie die Abtreibung nicht vornehmen, haben Sie ein sehr hohes Risiko, dass alle sieben Föten nicht überleben oder schwer geschädigt sind.

Wie angemessen ist es für Sie, drei Föten abtreiben zu lassen?

1. Assisted suicide state

Sie sind Mitglied des kanadischen Senats im Jahr 2017 und wirken bei der Verabschiedung von neuen Gesetzen mit. In Kanada steigen die Kosten für die Gesundheitsversorgung wegen der älter werdenden Bevölkerung immer weiter. Es gibt neue Untersuchungen, die zeigen, dass die Legalisierung von aktiver Sterbehilfe diese Kosten enorm senken würde. Sie überlegen deshalb aktive Sterbehilfe zu erlauben, wenn ein sterbenskranker Patient eine Lebenserwartung von weniger als 6 Monaten hat. Diese Patienten können sich dann, wenn sie es möchten, von einem Arzt durch eine Überdosis Beruhigungsmittel töten lassen. Das würde nach Schätzungen jährliche Kosteneinsparungen von 139 Millionen Dollar in Kanada mit sich bringen. Dieses Geld könnte für andere Gesundheitsleistungen genutzt werden.

Wenn Sie aktive Sterbehilfe erlauben, könnten also viel mehr Menschen besser behandelt werden, wodurch sich deren Lebensqualität und Lebenserwartung verbessern würde. Gleichzeitig würden Sie damit aber Tötung legalisieren. Wenn Sie aktive Sterbehilfe nicht erlauben, wird im Gesundheitssystem weiter stark gespart werden müssen.

Wie angemessen ist es für Sie, das Gesetz zu ändern und aktive Sterbehilfe zu erlauben?

1. Atom bomb

Sie sind Physiker, leben seit einigen Jahren in den USA und haben wichtige Arbeiten zur Erforschung von Kernenergie geleistet. Im Jahr 1939 verdichten sich Gerüchte, dass das Nazi-Regime mit dem Bau der ersten Atombombe beschäftigt ist. Sie gehen davon aus, dass Deutschland seine Atombombe nicht einsetzen wird, wenn bekannt ist, dass die USA ebenfalls eine Atombombe besitzen. Deshalb überlegen Sie, den amerikanischen Präsidenten aufzufordern, auch auf Seiten der USA eine Atombombe bauen zu lassen und selbst daran mitzuarbeiten.

Wenn Sie dafür sorgen, dass eine amerikanische Atombombe gebaut wird, könnten Sie den Einsatz der deutschen Atombombe verhindern. Gleichzeitig würden Sie damit aber dazu beitragen, die größte bisher denkbare Massenvernichtungswaffe zu bauen.

Wie angemessen ist es für Sie, den Präsidenten zum Bau der Atombombe auffordern?

**Factual–Saving (prescriptive norm, i.e., action coincides with deontological option)**

1. Medicine costs

Sie sind im Jahr 2013 belgische Gesundheitsministerin. Ein 7-jähriger Junge, der an einer sehr seltenen Immunkrankheit leidet, bittet Sie um Kostenerstattung für ein Medikament. Ohne das Medikament regelmäßig einzunehmen wird der Junge sterben. Das Medikament wird bisher nicht vom Gesundheitssystem bezahlt und ist so teuer, dass die Familie es nicht mehr lange schaffen wird, genug Geld zu beschaffen (mehr als 200.000 Euro pro Jahr). Deshalb wird der Junge sterben, wenn Sie die Kostenerstattung verweigern.

Wenn Sie die Kosten für das Medikament nicht erstatten, wird der Junge sehr wahrscheinlich in den nächsten Jahren sterben. Wenn Sie die Kosten erstatten, fehlt aber viel Geld im Gesundheitssystem, was dazu führen wird, dass andere Behandlungen nicht finanziert werden können. Also wird die Kostenerstattung für den Jungen wahrscheinlich dazu führen, dass mehrere andere Menschen sterben.

Wie angemessen ist es für Sie, die Kostenerstattung für den Jungen übernehmen (um damit sein Leben zu retten)?

1. RAF

Sie sind deutscher Bundeskanzler im Jahr 1977 und die RAF (Rote-Armee-Fraktion) hat den Präsidenten der Bundesvereinigung der Deutschen Arbeitgeberverbände entführt. Die RAF fordert als Austausch für das Leben des Präsidenten die Freilassung von elf inhaftierten RAF-Mitgliedern. In einer früheren Entführung (1975) hatte die RAF erfolgreich fünf inhaftierte RAF-Mitglieder freigepresst. Vier von diesen haben anschließend erneut Terroranschläge begangen und dabei Menschen getötet.

Wenn sie in die Forderungen der RAF einwilligen, werden elf frühere Terroristen wieder freigelassen und wahrscheinlich wieder Anschläge durchführen, wodurch viele Menschen sterben könnten. Wenn sie die Forderungen ablehnen, wird die Geisel getötet werden.

Wie angemessen ist es für Sie, die früheren Terroristen freizulassen?

(Wie angemessen ist es für Sie, das Leben der Geisel zu retten, selbst wenn durch die Freilassung der Terroristen mehr Menschen sterben?)

1. Mare nostrum

Sie sind Mitglied des Außenministeriums Großbritanniens im Jahr 2014. Viele Kriegs- und Wirtschaftsflüchtlinge aus Nordafrika wollen über das Mittelmeer nach Europa. Die EU finanziert das Rettungsprogramm „Mare Nostrum“, das schon 150.000 Menschen in Seenot gerettet hat. Illegale Banden, sogenannte Schlepper, machen sich das zunutze: Sie geben gegen hohe Bezahlung seeuntüchtige Boote an Flüchtlinge und verweisen darauf, dass diese auf dem Meer gerettet würden. Dadurch sind 2014 allerdings über 3.000 Menschen ertrunken oder verdurstet, weil sie nicht rechtzeitig gerettet werden konnten. Ihre Regierung denkt darüber nach, das Rettungsprogramm abzubrechen, um den Schleppern entgegenzuwirken und Flüchtlinge abzuschrecken. Dadurch würden kurzfristig mehr Menschen auf dem Meer sterben; langfristig kämen aber vermutlich weniger über das Mittelmeer, wodurch es in Zukunft weniger Tote gäbe.

Wenn Sie die Rettungsaktion fortführen, müssen erstmal weniger Flüchtlinge sterben, aber langfristig sterben weiterhin Tausende im Meer. Wenn Sie die Rettungsaktionen abbrechen, werden Flüchtlinge abgeschreckt und Schlepperbanden nicht mehr unterstützt, jedoch würden Flüchtende sterbend auf dem Meer zurückgelassen.

Wie angemessen ist es für Sie, die Rettungsaktionen fortzuführen (um Flüchtlinge auf dem Mittelmeer zu retten)?

1. Rwanda

Sie sind Projektleiter eines Entwicklungshilfe-Teams als es 1994 überall in Ruanda zu Übergriffen der Hutu-Volksgruppe auf die Tutsi-Volksgruppe kommt. Die Familie eines Ihrer Mitarbeiter ist zu Ihnen ins Entwicklungshilfezentrum geflüchtet, als noch ein weiterer Mitarbeiter eintrifft. Dieser berichtet, dass seine gesamte Familie sowie einer Ihrer Mitarbeiter von einer bewaffneten Menschenmenge getötet wurden, weil sie der Tutsi-Volksgruppe angehören. Kurz danach versammelt sich die bewaffnete Menschenmenge vor dem Entwicklungshilfezentrum. Der Verhandlungsführer droht, alle Menschen im Haus zu töten, wenn Sie dem soeben eingetroffenen Mitarbeiter Unterschlupf gewähren.

Wenn Sie den Mitarbeiter verstecken, dann wird die Menschenmenge vermutlich das Entwicklungshilfezentrum angreifen und alle Menschen darin töten. Wenn Sie den Mitarbeiter an die Menschenmenge übergeben, werden diese Ihren Mitarbeiter töten, aber alle anderen Menschen in Ihrer Obhut werden verschont.

Wie angemessen ist es für Sie, den geflüchteten Mitarbeiter im Entwicklungshilfezentrum zu schützen?

1. Sahara ransom

Sie sind 2003 deutscher Bundeskanzler und müssen entscheiden, ob Sie mehrere Millionen Euro Lösegeld zahlen. In der Sahara wurden zehn Geiseln von Terroristen gefangen genommen. Die Geiselnahme dauert schon fast ein halbes Jahr an und eine Frau ist bereits gestorben. Aus vergangenen Entführungen ist bekannt, dass Terroristen sich durch Lösegelder finanzieren und mit den Geldern wieder Waffen kaufen und damit viele Menschen töten. Außerdem werden durch die Lösegeldzahlung zukünftige Touristen zur Beute für Geiselnehmer.

Wenn Sie das Lösegeld zahlen, können Sie die Geiseln retten. Wenn Sie das Lösegeld nicht zahlen, werden die Geiseln getötet, aber Sie vermeiden damit, dass sich zukünftiger Terrorismus durch weitere Geiselnahmen finanziert und retten damit wahrscheinlich mehr Menschenleben.

Wie angemessen ist es für Sie, das Lösegeld zu bezahlen?

(Wie angemessen ist es für Sie, die Geiseln zu retten, auch wenn Sie damit Terroristen Geld geben?)

1. Aid for Syria

In Syrien herrscht seit Jahren Bürgerkrieg zwischen dem syrischen Regime und Rebellen. 2016 sind die Zustände katastrophal, die Bevölkerung hat weder genügend Nahrungsmittel noch medizinische Versorgung. Sie sind Mitarbeiter der UN und verantwortlich für die Auszahlung von Hilfsgeldern. Ihre Vorgesetzten haben mehrere zehn Millionen für Syrien bewilligt, die an lokale Hilfsorganisationen gehen sollen. Alle lokalen Hilfsorganisationen werden allerdings vom syrischen Regime kontrolliert. Das Regime steht unter dem dringenden Verdacht, Hilfsgüter bevorzugt in eigenen Gebieten zu bringen und sogar Hilfsgelder zu veruntreuen, um den Krieg zu finanzieren. Deswegen geben andere Organisationen keine Hilfszahlungen mehr nach Syrien, denn sie wollen das syrische Regime nicht unterstützen. Dennoch könnten die Gelder vielen Notleidenden helfen und Leben retten.

Wenn Sie die Hilfsgelder freigeben, helfen Sie bis zu Millionen Menschen unterstützen damit aber das Syrische Regime. Wenn Sie die Zahlungen verweigern, erhalten bis zu Millionen Menschen so gut wie keine Hilfsgüter mehr.

Wie angemessen ist es für Sie, die Hilfsgelder für das syrische Regime freizugeben (um Kriegsopfern zu helfen)?

**Factual Other proscriptive norms (action coincides with utilitarian option)**

1. Rescue torture

Sie sind Polizeipräsident und ermitteln im Jahr 2002 wegen der Entführung eines Bankierssohnes. Sie haben einen Verdächtigen festgenommen, der das Lösegeld abgeholt hat. Der Verdächtige hat im Laufe des Verhörs gestanden, dass er den Jungen entführt hat, weigert sich jedoch, sein Versteck bekannt zu geben. Sie haben den Verdächtigen seit 2,5 Tagen unter Beobachtung und befürchten, dass der Junge bald verdursten könnte. Deshalb überlegen Sie den Verdächtigen durch Drohungen und körperliche Gewalt dazu zu bringen, das Versteck der Geisel zu nennen.

Wenn Sie die Gesetze brechen indem Sie den Verdächtigen foltern lassen, wird er wahrscheinlich das Versteck der Geisel verraten. Wenn Sie sich an die Gesetze halten und den Verdächtigen nicht foltern, wird er vielleicht noch tagelang das Versteck der Geisel verschweigen, die dann sterben könnte.

Wie angemessen ist es, dass Sie den Verdächtigen foltern (damit er das Versteck der Geisel verrät)?

1. War torture

Sie sind 1940 der Leiter einer Einrichtung zum Verhör deutscher Kriegsgefangener in Großbritannien. In Ihre Einrichtung kommen Gefangene, die möglicherweise wichtige Informationen haben. Durch solche Informationen könnten tausende Leben gerettet werden. Aber nur sehr wenige Gefangenen haben wirklich relevante Informationen. Für Großbritannien steht gerade alles auf dem Spiel: Frankreich wird von den Deutschen überrollt und die deutsche Luftwaffe bombardiert britische Städte. Ihre Vorgesetzten verlangen schnelle Ergebnisse – notfalls mit allen Mitteln. Daher überlegen Sie, alle Gefangenen systematisch foltern zu lassen, um schnellstmöglich wichtige Informationen zu bekommen. Ein solches Vorgehen würde allerdings klar gegen die Genfer Konventionen verstoßen, nach denen Großbritannien verpflichtet ist, Personen zu schützen, die nicht mehr an den Kampfhandlungen teilnehmen.

Wenn Sie die Gefangenen systematisch foltern lassen, können Sie sehr wahrscheinlich tausende Menschenleben retten. Wenn Sie den Befehl nicht geben, verlieren Sie womöglich lebensrettende Informationen.

Wie angemessen ist es für Sie, den Befehl zum Foltern zu geben (um tausende Leben zu retten)?

1. Rugby cannibalism

Sie sind 1972 Teil einer Rugbymannschaft und fliegen mit Ihren Kollegen sowie Freunden und Bekannten von Montevideo zu einem Freundschaftsspiel nach Santiago de Chile. Insgesamt sind 40 Passagiere an Bord. Das Flugzeug stürzt ab, wobei 15 Menschen sterben. Sie befinden sich in 4.000 Meter Höhe bei -30 Grad. Nach 10 Tagen hören Sie durch ein kleines Radio, dass die Suche nach Ihrem Flugzeug aufgegeben wurde. Mittlerweile besteht Ihre Gruppe nun nur noch aus 19 Überlebenden und Ihre Nahrungsmittel sind aufgebraucht. Nun überlegen Sie mit den Anderen, ob Sie die Überreste Ihrer toten Freunde und Bekannten essen sollen, womit Sie ihre Menschenwürde und die Totenruhe verletzen müssten.

Wenn Sie und der Rest der Gruppe das Menschenfleisch essen, werden Sie länger überleben und Ihre Chance erhöhen entdeckt zu werden oder Hilfe zu finden. Wenn Sie und der Rest der Gruppe das Menschenfleisch nicht essen, werden Sie verhungern.

Wie angemessen ist es für Sie, die Überreste Ihrer toten Freunde und Mitstreiter zu essen (um Ihre Chance auf Überleben zu erhöhen)?

1. Robin Hood

Sie sind 1999 Bankangestellter in einer Gemeinde in Süddeutschland. Sie haben zahlreiche Kunden, die Ihnen und Ihrem Geldinstitut vertrauen und treue Kunden sind. Darunter sind allerdings einige Kunden, die Schulden haben, und manche denen Sie Kredite verweigern müssen. Beratungsgespräche mit solchen Kunden belasten Sie sehr. So bittet Sie einer dieser Kunden verzweifelt um einen Kredit für seine Familie, da diese sonst finanziell am Ende wäre. Sie könnten unbemerkt Einzahlungen und Wertpapieranlagen anderer Kunden auf sein Konto umleiten, um seine Kreditwürdigkeit wiederherzustellen; dann könnten Sie dem Kunden mit einem Kredit helfen. Auch anderen verschuldeten Kunden könnten Sie so unbemerkt helfen. Jedoch würden Sie damit das Vertrauen von vermögenderen Kunden und Vorgesetzten missbrauchen und sich des Betrugs schuldig machen.

Wenn Sie einen Teil des Geldes Ihrer vermögenden Kunden umbuchen, können Sie verschuldeten Kunden helfen. Wenn Sie alle Ihre Kunden gemäß den Vorschriften betreuen, dann gehen viele der verschuldeten Kunden und deren Familien pleite.

Wie angemessen ist es für Sie, geringe Geldbeträge von vermögenden Kunden umzubuchen (um Kunden in finanzieller Not zu helfen)?

1. Knee operations

Sie sind 2017 Arzt in einem Krankenhaus einer Kreisstadt in Süddeutschland. Damit ein Krankenhaus Knieoperationen von der Krankenkasse erstattet bekommt, müssen pro Jahr mindestens 50 Knieoperationen durchgeführt werden. Ihre Klinik hat bis Dezember nur 48 Knieoperationen durchgeführt, sodass Sie keine einzige Knieoperation bezahlt bekommen würden. Da die Klinik schon in den letzten Jahren rote Zahlen geschrieben hat und jetzt weitere 336.000 Euro Schulden durch die Knieoperationen hinzukommen, steht das Krankenhaus vor dem Bankrott. Ohne dieses Krankenhaus kann allerdings für die Region keine schnelle Notfallversorgung gewährleistet werden, was früher oder später zu zusätzlichen Todesfällen führen wird. Die einzige Möglichkeit zur Rettung der Klinik besteht darin, zwei Knieoperationen durchzuführen, die medizinisch nicht nötig sind.

Wenn Sie zwei nicht notwendigen Knieoperationen durchführen, könnte die Klinik vor dem Bankrott gerettet werden und die Region wäre weiterhin gut medizinisch versorgt; allerdings operieren Sie dann Patienten, die ohne Operation besser therapiert werden könnten. Wenn Sie die Knieoperationen nicht durchführen, steht die Klinik vor dem Bankrott, was früher oder später zu Todesfällen führen wird.

Wie angemessen ist es für Sie, die beiden therapeutisch nicht notwendigen Operationen durchzuführen?

1. Organ market

Sie leben in Israel und sind seit einem Nierenversagen vor 4 Jahren auf Dialyse angewiesen. In Ihrer Familie ist niemand als Nierenspender für Sie geeignet und die offizielle Warteliste ist so lang, dass viele Menschen sterben, weil sie keine Spenderniere erhalten. Sie erleiden einen kleineren Herzinfarkt, weshalb Sie auf der Warteliste nach unten gestuft werden. Damit ist es praktisch ausgeschlossen, dass Sie eine Spenderniere erhalten werden. Sie überlegen eine Niere auf dem Schwarzmarkt zu kaufen. Menschen aus armen Ländern verkaufen Nieren, die zahlungskräftigen Kranken transplantiert werden. Die Gesundheitsrisiken sind dabei gering. Auch der Spender kann mit einer Niere weiterleben, wird allerdings gesundheitlich eingeschränkt und in Zukunft anfälliger sein. Organhandel ist gut organisiert aber illegal, weil damit die Not armer Menschen dazu ausgenutzt wird, ihre eigene Gesundheit aufs Spiel zu setzen.

Wenn Sie keine Niere kaufen, werden Sie sicher in ein paar Jahren sterben. Wenn sie eine Niere kaufen, werden wahrscheinlich sowohl Sie als auch der Spender mehrere Jahrzehnte lang gut leben können. Gleichzeitig nutzen Sie damit aber die Armut des Spenders aus.

Wie angemessen ist es, dass Sie sich eine Niere vom Schwarzmarkt kaufen (um zu überleben)?

1. Tax fraud detection

Sie sind Finanzminister von Nordrhein-Westfalen im Jahr 2015. Ihnen wurde eine CD zum Kauf angeboten, die Daten über Personen mit Konten bei luxemburgischen Banken und Kreditinstituten enthält. Die Daten würden Informationen über Steuerhinterzieher liefern. Die CD wurde von Hackern erstellt, die sich illegal Zugang zu den Computern der luxemburgischen Bank geschaffen haben, die Daten scheinen jedoch zuverlässig zu sein. Auf der CD sind bis zu 50.000 Vorgänge enthalten.

Wenn Sie die CD kaufen, nutzen Sie illegal beschaffte Informationen und belohnen die Hacker für ihren Diebstahl, erhalten dafür aber Steuernachzahlungen und Bußgelder in Millionenhöhe. Wenn sie die CD nicht kaufen, entgehen Ihnen Steuergeldnachzahlungen und Bußgelder.

Wie angemessen ist es für Sie, die CD zu kaufen (um die Zahlungen zu erhalten)?

1. Ashley treatment

Sie sind Arzt und Spezialist für Fragen der medizinischen Ethik und arbeiten im Jahr 2004 in Seattle, als die Eltern eines 6-jährigen Mädchens auf Sie zukommen. Ihre Tochter leidet an statischer Enzephalopathie, kann weder gehen noch sitzen und ist und bleibt geistig auf dem Stand eines 3-monatigen Babys. Noch können die Eltern sie gut häuslich pflegen, allerdings befindet sich das Mädchen im Wachstum. Um zu verhindern, dass sie zu schwer wird, wollen die Eltern ihr Wachstum anhalten und ihre Sexualentwicklung verhindern, indem sie Uterus und Brustgewebe entfernen sowie eine hoch dosierte Östrogentherapie verabreichen lassen. Diese Behandlungen sind gesundheitlich nicht notwendig und das Kind kann nicht selbst entscheiden. Die Behandlung würde also das Recht des Kindes auf körperliche Unversehrtheit verletzen.

Wenn Sie die Eingriffe nicht vornehmen, kann das Mädchen bald nicht mehr von seinen Eltern gepflegt werden und somit nicht im familiären Umfeld bleiben.

Wie angemessen ist es für Sie, die Eingriffe bei dem Kind vorzunehmen?

1. Test tube sibling

Sie sind Richter eines britischen Berufungsgerichts im Jahr 2003 und sollen über eine künstliche Befruchtung entscheiden. Ein Junge mit Beta-Thalassämie, einer tödlichen Bluterkrankung, kann nur durch eine Stammzellen- oder Knochenmarkspende gerettet werden. Bisher wurde kein Spender gefunden. Nun wollen die Eltern mit künstlicher Befruchtung ein weiteres Kind zeugen, mit dessen Stammzellen ihr kranker Sohn gerettet werden soll. Aus den Embryonen soll nur ein genetisch passender ausgewählt und in die Gebärmutter eingepflanzt werden. Verlaufen die Schwangerschaft und die Transplantation der Stammzellen aus der Nabelschnur erfolgreich, liegen die Chancen für eine völlige Heilung des kranken Kindes bei über 90%. Gleichzeitig würde damit aber ein Kind als Organspender gezüchtet.

Wenn Sie diese künstliche Befruchtung erlauben, kann das kranke Kind mit großer Wahrscheinlichkeit geheilt werden. Wenn Sie diese künstliche Befruchtung nicht erlauben, wird das kranke Kind sterben, wenn weiterhin kein geeigneter Spender gefunden wird.

Wie angemessen ist es für Sie, die künstliche Befruchtung zu erlauben?

1. Lion hunt

Sie arbeiten im Jahr 2006 bei der Naturschutzbehörde „International Union for Conservation of Nature in Tansania“. Sie sind für den Schutz bedrohter Tierarten zuständig, unter anderem für Löwen, von denen es in freier Wildbahn immer weniger gibt. Forschung hat gezeigt, dass die größer werdenden Vieh- und Ackerflächen der Hauptgrund für das Aussterben der Löwen ist. Um den Lebensraum der Löwen längerfristig zu gewährleisten, möchten Sie Land kaufen und unter Naturschutz stellen. Sie haben allerdings trotz großer Anstrengungen und vieler Spendenkampagnen kaum Geld zur Verfügung. Deshalb denken Sie darüber nach, einzelne der bedrohten Löwen zu töten und die Felle für 60.000 Dollar je Stück zu verkaufen.

Wenn Sie einige Löwen töten, hätten Sie viel Geld, um damit Land für den Schutz der verbleibenden Löwen zu kaufen. Wenn Sie keine Löwen töten, haben Sie keine Möglichkeit Land unter Naturschutz zu stellen.

Wie angemessen ist es für Sie, einige Löwen zu töten (um mit dem Geld Flächen unter Naturschutz stellen zu können)?

**Factual Other prescriptive norms (action coincides with deontological option)**

1. Bishop

Sie sind katholischer Erzbischof von Utrecht, als die deutschen Besatzer im Jahr 1942 beginnen niederländische Juden zu deportieren. Sie wissen, dass den Deportierten großes Leid widerfährt, auch wenn Sie nicht wissen, was genau passiert. Durch ein stilles Abkommen mit den Deutschen könnten Sie sicherstellen, dass wenigstens die zahlreichen Christen jüdischer Abstammung verschont bleiben. Andererseits überlegen Sie, die Deportationen öffentlich zu verurteilen. Sie riskieren damit allerdings, dass auch die bislang verschonten Christen jüdischer Abstammung (darunter auch Mitglieder Ihrer Gemeinde) deportiert werden.

Wenn Sie zu den Deportationen weiter öffentlich schweigen, werden zwar weiterhin niederländischen Juden deportiert, doch die Christen jüdischer Abstammung bleiben verschont. Wenn Sie die Deportationen öffentlich verurteilen, dann bringen Sie das Leben aller Christen jüdischer Abstammung in Gefahr, doch kommen Ihrer moralischen Pflicht nach, auf die unrechtmäßige Deportationen von Menschen aufmerksam zu machen.

Wie angemessen ist es, dass Sie die Deportation öffentlich anprangern?

(Wie angemessen ist es, dass Sie das Leben der Christen jüdischer Abstammung in Gefahr bringen, um auf die Deportationen aufmerksam zu machen?)

1. Trail of Tears

Sie sind führendes Mitglied des Stammes der Cherokee, als der US-Kongress 1830 beschließt, alle Ureinwohner im Süden der Vereinigten Staaten, also auch Ihren Stamm, aus ihren angestammten Siedlungsgebieten nach Westen umzusiedeln. Sie waren bislang ein Gegner von Umsiedlungsverhandlungen, verstoßen diese eindeutig gegen die Menschenrechte, die die US-Verfassung allen US-Bürgern und den Ureinwohnern garantiert. Auch der Supreme Court hat bereits zu Gunsten Ihres Stammes entscheiden. Doch Sie wissen, dass die weißen Siedler und der US-Präsident eine Weigerung zur Umsiedelung nicht akzeptieren, sodass es zum Krieg mit den weit überlegenen US-Streitkräften kommen wird.

Wenn Sie sich für eine freiwillige Umsiedelung einsetzen, ist die Wahrscheinlichkeit hoch, dass Ihr Stamm ohne Blutvergießen abziehen wird, doch Sie geben damit unwiderruflich Ihr traditionelles Stammesgebiet auf. Wenn Sie sich weiterhin gegen eine Umsiedelung aussprechen, dann wird es Krieg mit den US-Streitkräften geben und viele Ihrer Stammesangehörige werden sterben.

Wie angemessen ist es für Sie, weiterhin Widerstand gegen eine Umsiedelung zu leisten (um Ihren Stammestraditionen weiter nachgehen zu können, auch wenn das zu einem Krieg mit vielen Toten führen wird)?

1. Veterinarian

Sie sind Tierärztin auf der Ostsee-Insel Riems, wo hochansteckende Krankheitserreger an Tieren erforscht werden. Sie fügen täglich Tieren Leid zu, indem Sie sie mit tödlichen Krankheiten infizieren. Sie wissen, dass diese Arbeit wichtige Erkenntnisse liefert, die andere Tiere rettet. Zudem sind Sie eine Spezialistin auf Ihrem Gebiet und können mit Ihrem Wissen mehr bewirken als andere Tierärzte. Doch Sie sind Tierärztin geworden, um Tiere zu heilen, und denken darüber nach, Ihre Stelle zu kündigen.

Wenn Sie Ihre Stelle kündigen, sterben dort deutlich weniger Tiere, doch werden auch weniger Erkenntnisse über gefährliche und tödliche Tierkrankheiten erlangt, was für viele andere Tiere den Tod bedeutet. Wenn Sie weiterhin täglich Tiere mit Krankheitserregern infizieren, fügen Sie weiterhin den Tieren in Ihrer Obhut Leid zu, doch retten durch Ihre Forschung wahrscheinlich vielen anderen Tieren das Leben.

Wie angemessen ist es für Sie, Ihre Stelle zu kündigen (um damit Tieren kein unnötiges Leid mehr anzutun, auch wenn Sie damit keine Forschungsergebnisse mehr erhalten, die andere Tiere retten würden)?

1. Hogesa

Sie sind Verwaltungsrichter der Stadt Köln im Jahr 2015. Eine rechts-politische Gruppierung namens "Hogesa" (Hooligans gegen Salafisten) möchte eine Demonstration gegen islamistischen Extremismus in der Innenstadt abhalten. Freie Meinungsäußerung und Versammlungsfreiheit sind unveräußerliche Grundrechte und damit ein hohes Gute. Allerdings ist es bei vergangenen Demonstrationen dieser Gruppierung immer wieder zu Gewalt gegen Polizisten und Ausländer gekommen.

Wenn Sie die Demonstration verbieten, nehmen Sie den Menschen der Gruppierung ihr Recht auf freie Meinungsäußerung. Wenn Sie die Demonstration erlauben, besteht die Gefahr, dass Menschen verletzt werden.

Wie angemessen ist es für Sie, die Demonstration zu erlauben (um die Versammlungsfreiheit der Gruppierung zu gewährleisten, auch wenn damit Menschen verletzt werden)?

1. Mass rape

Sie sind Ermittlungsrichter in Berlin Anfang der 90er Jahre. Auf einem Volksfest saß eine Gruppe von acht betrunkenen Männern zusammen, als die 17-jährige Kellnerin hinter einem Vorhang von der Gruppe vergewaltigt wurde. Einer der Männer machte nicht mit und rief die Polizei. Als diese kam, fanden sie das Mädchen nackt, verletzt und bewusstlos unter der Bühne. Die Ermittlungen haben keine Erkenntnisse gebracht, da die Männer schweigen, das Mädchen keine genauen Aussagen treffen kann und die Spuren auf ihrem Körper unbrauchbar waren.

Wenn Sie alle Männer in Untersuchungshaft nehmen, wird der Unschuldige zu Unrecht ins Gefängnis gesperrt. Wenn Sie die Männer gehen lassen, werden sieben Vergewaltiger freigelassen [und begehen möglicherweise weitere Straftaten].

Wie angemessen ist es für Sie, alle Männer frei zu lassen (um den Unschuldigen vor ungerechter Strafe zu bewahren, selbst wenn dadurch viele Schuldige freikommen)?

1. Endowment

Nach langjähriger Haft will ein Kindesentführer und -mörder im Jahr 2006 eine Stiftung gründen für Kinder, die Opfer eines Gewaltverbrechens wurden. Sie sind Präsident der Behörde, die über die Einrichtung der Stiftung entscheidet. Der Stifter würde dabei ein Startkapital von 25.000€ mitbringen. Jedoch soll die Stiftung nach dem Täter benannt werden und es ist unklar, ob dieser aus Reue handelt oder nur seinen Namen reinwaschen will. Die Familie des damals ermordeten Kindes sowie Unterstützer lehnen die Stiftung ab, sie empfinden die Stiftung als sittenwidrig und als Herabwürdigung, Verhöhnung und Ausnutzung des Opfers.

Wenn Sie die Stiftung ablehnen geht Startkapital verloren. Wenn Sie die Stiftung genehmigen, können Opfer von Gewaltverbrechen durch Sammeln von Spenden, Sponsoren und das Startkapital unterstützt werden.

Wie angemessen ist es für Sie, die Stiftung abzulehnen (um damit die Herabwürdigung des Opfers zu verhindern)?
