# Supplementary material for: Deontology and Utilitarianism in Real Life: A Set of Moral Dilemmas Based on Historic Events
Source: Pers Soc Psychol Bull. 2022 Jun 24;49(10):1511–28. doi: 10.1177/01461672221103058 (PMC10478346; doi:10.1177/01461672221103058)
Supplement: sj-docx-2-psp-10.1177_01461672221103058 – Supplemental material for Deontology and Utilitarianism in Real Life: A Set of Moral Dilemmas Based on Historic Events [file sj-docx-2-psp-10.1177_01461672221103058.docx]

**Supplemental Materials for:**

**Real-life Dilemmas: Scenarios Contrasting Deontology and Utilitarianism Based on Historic Situations**

**Additional Results Study 1**

In addition to the analysis reported in the main document, we compared judgments of trolley-type dilemmas and the type of factual dilemmas that are most similar to trolley-type dilemmas, that is, factual dilemmas where the utilitarian action consists in killing humans. In contrast to the main analysis, as the number of factual–killing dilemmas differed considerably between participants, these analyses were performed with dilemmas as units of analysis. The factual killing dilemmas were judged to be more typical (*M* = 5.00, *SD* = 0.42) than trolley-type dilemmas (*M* = 3.91, *SD* = 0.47), *t*(29) = 6.84, *p* < .001, *d* = 2.46, 95% CI [1.50, 3.39]. Moreover, factual–killing dilemmas were also judged to be more plausible (*M* = 4.32, *SD* = 0.42) than trolley-type dilemmas (*M* = 3.65, *SD* = 0.43), *t*(29) = 4.40, *p* < .001, *d* = 1.58, 95% CI [0.76, 2.38]; factual–killing dilemmas were judged to be less absurd (*M* = 3.19, *SD* = 0.61) than trolley-type dilemmas (*M* = 4.68, *SD* = 0.66), *t*(29) = 6.55, *p* < .001, *d* = 2.35, 95% CI [1.42, 3.27]. Thus, the results concerning realism replicated when using only factual–killing dilemmas. Additionally, factual–killing dilemmas elicited somewhat similar judgments (*M* = 3.99, *SD* = 1.03) to trolley-type dilemmas (*M* = 3.54, *SD* = 1.02), *t*(29) = 1.23, *p* = .229, *d* = 0.44, 95% CI [-0.28, 1.15].

Previous research found that self-benefit (the person making the judgment is in the group that benefits from the utilitarian action) and the victim dying in any case (in Table 1 & 2 referred to as “suffering subset of the benefitted”) both increase utilitarian judgments (e.g., Moore et al., 2008). Approximately half of the factual killing dilemmas and the trolley-type dilemmas involve neither self-benefit nor the victim’s dying in any case. Arguably, these dilemmas offer the purest comparison between the two dilemma classes. Including only these dilemmas leads to qualitatively identical results to the ones reported above. Specifically, the factual killing dilemmas were judged to be more typical (*M* = 5.18, *SD* = 0.30) than trolley-type dilemmas (*M* = 3.65, *SD* = 0.49), *t*(11) = 6.59, *p* < .001, *d* = 3.67, 95% CI [1.78, 5.50], factual–killing dilemmas were judged to be more plausible (*M* = 4.19, *SD* = 0.23) than trolley-type dilemmas (*M* = 3.35, *SD* = 0.46), *t*(11) = 4.08, *p* = .002, *d* = 2.27, 95% CI [0.80, 3.68]; and factual–killing dilemmas were judged to be less absurd (*M* = 2.83, *SD* = 0.57) than trolley-type dilemmas (*M* = 5.06, *SD* = 0.67), *t*(11) = 6.40, *p* < .001, *d* = 3.56, 95% CI [1.71, 5.36]. Moral judgments did not differ significantly for factual killing dilemmas (*M* = 4.03, *SD* = 1.01) and trolley-type dilemmas (*M* = 3.17, *SD* = 1.16), *t*(11) = 1.41, *p* = .186, *d* = 0.79, 95% CI [-0.37, 1.91]. Moreover, including these variables (self-benefit and subset) as covariates does not significantly alter the results reported in Table 5, see https://osf.io/cg5tq/. Thus, the present results are not driven by confounds between realism and self-benefit or the victim’s suffering in any case. Instead, the observed differences are driven by greater realism for factual compared to trolley-type dilemmas.

The correlation analysis reported in the main text (see Table 6) included all dilemmas evaluated in Study 1. Additionally, it could be interesting to examine these correlations for each type of dilemma separately. To increase the N, we used three separate dilemma types, trolley-type dilemmas (Table S1), factual dilemmas where the action coincides with the utilitarian option (both when this action involves killing and when it involves other norm transgressions, see Table S2), and factual dilemmas where the action coincides with the deontological option (both when this action involves saving and when it involves other norms, see Table S3).

**Table S1**

*Correlation Matrix for Evaluations of Trolley-Type Dilemmas (N = 15), Study 1*

|  | Typicality | Plausibility | Absurdness | Arousal | Valence | Difficulty | Response Time |
| --- | --- | --- | --- | --- | --- | --- | --- |
| Judgment | .186 | .362 | -.310 | -.149 | .412 | .470 | .361 |
| Typicality |  | .841^**^ | -.977^**^ | .540^*^ | -.455 | .352 | -.017 |
| Plausibility |  | - | -.870^**^ | .428 | -.343 | .549^*^ | .232 |
| Absurdness |  |  | - | -.452 | .350 | -.392 | -.020 |
| Arousal |  |  |  | - | -.849^**^ | .631^*^ | .180 |
| Valence |  |  |  |  | - | -.424 | .013 |
| Difficulty |  |  |  |  |  | - | .289 |

*Note*. Typicality, plausibility, and absurdness: 1 = not at all, 7 = completely; Judgment: 1 = not at all appropriate, 7 = completely appropriate; Arousal: 1 = not at all arousing, 7 = very arousing; Valence: 1 = very negative feelings, 7 = very positive feelings; Decision difficulty: 1 = not difficult, 7 = very difficult

^*:^ *p* < .05. ^**^ *p* < .01.

**Table S2**

*Correlation Matrix for Evaluations of Factual Dilemmas where Action Coincides with Utilitarianism (N = 26), Study 1*

|  | Typicality | Plausibility | Absurdness | Arousal | Valence | Difficulty | Response Time |
| --- | --- | --- | --- | --- | --- | --- | --- |
| Judgment | .333 | .645^**^ | -.476^*^ | -.136 | .243 | .251 | .185 |
| Typicality |  | .515^**^ | -.912^**^ | -.085 | .265 | .026 | .149 |
| Plausibility |  | - | -.547^**^ | .149 | -.045 | .360 | .026 |
| Absurdness |  |  | - | .243 | -.402^*^ | -.032 | -.271 |
| Arousal |  |  |  | - | -.909^**^ | .621^**^ | -.139 |
| Valence |  |  |  |  | - | -.612^**^ | .189 |
| Difficulty |  |  |  |  |  | - | .282 |

*Note*. Typicality, plausibility, and absurdness: 1 = not at all, 7 = completely; Judgment: 1 = not at all appropriate, 7 = completely appropriate; Arousal: 1 = not at all arousing, 7 = very arousing; Valence: 1 = very negative feelings, 7 = very positive feelings; Decision difficulty: 1 = not difficult, 7 = very difficult

^*:^ *p* < .05. ^**^ *p* < .01.

**Table S3**

*Correlation Matrix for Evaluations of Factual Dilemmas where Deontology Coincides with Action (N = 12), Study 1*

|  | Typicality | Plausibility | Absurdness | Arousal | Valence | Difficulty | Response Time |
| --- | --- | --- | --- | --- | --- | --- | --- |
| Judgment | .201 | .181 | -.031 | -.150 | .514 | .243 | .130 |
| Typicality |  | .637^*^ | -.905^**^ | -.023 | -.055 | .330 | -.147 |
| Plausibility |  | - | -.729^**^ | -.286 | .114 | .071 | -.282 |
| Absurdness |  |  | - | .142 | .116 | -.111 | .145 |
| Arousal |  |  |  | - | -.805^**^ | .740^**^ | .296 |
| Valence |  |  |  |  | - | -.518 | -.131 |
| Difficulty |  |  |  |  |  | - | .139 |

*Note*. Typicality, plausibility, and absurdness: 1 = not at all, 7 = completely; Judgment: 1 = not at all appropriate, 7 = completely appropriate; Arousal: 1 = not at all arousing, 7 = very arousing; Valence: 1 = very negative feelings, 7 = very positive feelings; Decision difficulty: 1 = not difficult, 7 = very difficult

^*:^ *p* < .05. ^**^ *p* < .01.

Recoding moral judgment so that greater values indicate more agreement with the utilitarian option, the correlation between the combined realism measure (consisting of typicality, plausibility, and reverse coded absurdness, Cronbach’s α = .91) and the utilitarianism score are depicted in Figure S1.

**Figure S1**

*Association between Realism Evaluations and Moral Judgments for Dilemma Types Separately, Study 1*


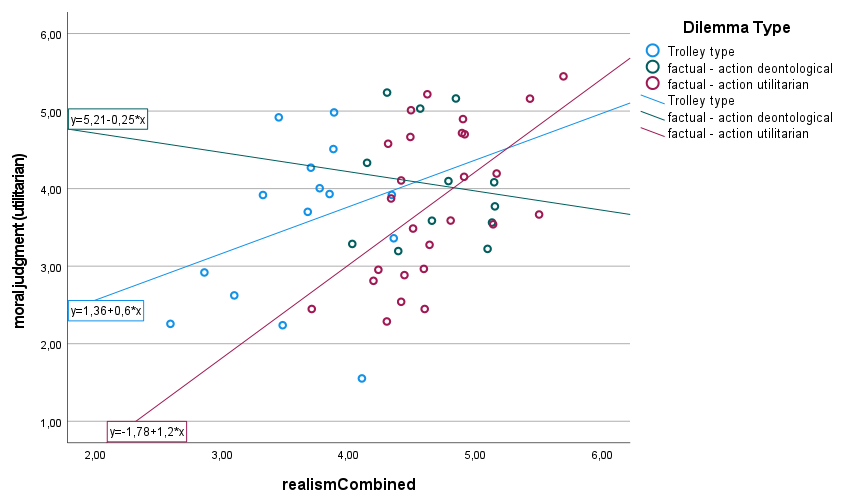


*Note*. Higher moral judgment values indicate more utilitarian judgments. Both scales ranged from 1 to 7.

**Additional Results Study 2**

In addition to the analysis reported in the main document, we compared judgments of trolley-type dilemmas and factual dilemmas that are most similar to trolley-type dilemmas, that is, factual dilemmas where the utilitarian action consists in killing humans. These analyses were performed with dilemmas as units of analysis. The factual killing dilemmas were judged to be more typical (*M* = 4.86, *SD* = 0.28) than trolley-type dilemmas (*M* = 4.35, *SD* = 0.36), *t*(34) = 4.61, *p* < .001, *d* = 1.54, 95% CI [0.78, 2.29]. Moreover, factual–killing dilemmas were also judged to be more plausible (*M* = 5.07, *SD* = 0.21) than trolley-type dilemmas (*M* = 4.78, *SD* = 0.25), *t*(34) = 3.77, *p* = .001, *d* = 1.26, 95% CI [0.53, 1.98]; factual–killing dilemmas were judged to be less absurd (*M* = 4.07, *SD* = 0.38) than trolley-type dilemmas (*M* = 4.68, *SD* = 0.40), *t*(34) = 4.58, *p* < .001, *d* = 1.54, 95% CI [0.78, 2.28]; and factual dilemmas were judged to be more easily taken seriously (*M* = 5.47, *SD* = 0.17) than trolley-type dilemmas (*M* = 5.03, *SD* = 0.31), *t*(30.47) = 5.42, *p* < .001, *d* = 1.71, 95% CI [0.93, 2.47]. Thus, the results concerning realism and participants’ taking the dilemmas seriously replicated when using only factual–killing dilemmas, which are structurally comparable to trolley-type dilemmas. Additionally, factual–killing dilemmas elicited more utilitarian judgments (*M* = 4.68, *SD* = 0.62) than trolley-type dilemmas (*M* = 4.15, *SD* = 0.63), *t*(34) = 2.51, *p* = .017, *d* = 0.84, 95% CI [0.15, 1.52].

Including only dilemmas where the victim is not going to die in any case and where there is no self-benefit for the person who makes the decision (see Study 1) leads to qualitatively identical results. Specifically, the factual killing dilemmas were judged to be more typical (*M* = 4.95, *SD* = 0.23) than trolley-type dilemmas (*M* = 4.09, *SD* = 0.27), *t*(12) = 6.26, *p* < .001, *d* = 3.38, 95% CI [1.65, 5.06]. Moreover, factual–killing dilemmas were also judged to be more plausible (*M* = 4.98, *SD* = 0.24) than trolley-type dilemmas (*M* = 4.63, *SD* = 0.25), *t*(12) = 2.65, *p* = .021, *d* = 1.43, 95% CI [0.21, 2.61]; factual–killing dilemmas were judged to be less absurd (*M* = 3.92, *SD* = 0.24) than trolley-type dilemmas (*M* = 4.96, *SD* = 0.28), *t*(12) = 7.22, *p* < .001, *d* =3.90, 95% CI [2.00, 5.74] and factual–killing dilemmas were taken more seriously (*M* = 5.44, *SD* = 0.13) than trolley-type dilemmas (*M* = 4.75, *SD* = 0.16), *t*(12) = 8.52, *p* < .001, *d* = 4.60, 95% CI [2.48, 6.68]. Moreover, moral judgments were more utilitarian for factual killing dilemmas (*M* = 4.74, *SD* = 0.65) and trolley-type dilemmas (*M* = 3.91, *SD* = 0.62), *t*(12) = 2.42, *p* = .032, *d* = 1.31, 95% CI [0.11, 2.46]. Moreover, including these variables (self-benefit and subset) as covariates does not significantly alter the results reported in Table 9, see https://osf.io/cg5tq/. Thus, the present results are not driven by confounds between realism and self-benefit or the victim’s suffering in any case. Instead, the observed differences can be attributed to greater realism for factual compared to trolley-type dilemmas.

As in Study 1, the correlations from the main text (Table 10) can be performed for each type of dilemma separately. To increase the N, we use only three dilemma types, trolley-type dilemmas (Table S4), factual dilemmas where the action coincides with the utilitarian option (both when this action involves killing and when it involves other norm transgressions, see Table S5), and factual dilemmas where the action coincides with the deontological option (both when this action involves saving and when it involves other norms, see Table S6).

**Table S4**

*Correlation Matrix for Evaluations of Trolley-type Dilemmas (N = 20), Study 2*

|  | Typicality | Plausibility | Absurdness | Taking Seriously | Arousal | Valence | Difficulty |
| --- | --- | --- | --- | --- | --- | --- | --- |
| Judgment | .259 | .628^**^ | -.626^**^ | .488^*^ | .164 | .516^*^ | .422 |
| Typicality |  | .638^**^ | -.784^**^ | .889^**^ | .392 | -.188 | .271 |
| Plausibility |  | - | -.784^**^ | .763^**^ | .410 | -.012 | .650^**^ |
| Absurdness |  |  | - | -.828^**^ | -.339 | -.180 | -.367 |
| Taking Seriously |  |  |  | - | .391 | .018 | .298 |
| Arousal |  |  |  |  | - | -.058 | .433 |
| Valence |  |  |  |  |  | - | .091 |

*Note*. Typicality, plausibility, absurdness, taking seriously: 1 = strongly oppose, 7 = strongly support; Arousal: 1 = not at all arousing, 7 = very arousing; Valence: 1 = very negative feelings, 7 = very positive feelings; Difficulty: 1 = not difficult, 7 = very difficult; Moral judgment: 1 = completely inappropriate, 7 = completely appropriate.

^*:^ *p* < .05. ^**^ *p* < .01.

**Table S5**

*Correlation Matrix for Evaluations of Factual Dilemmas where Action Coincides with Utilitarianism (N = 26), Study 2*

|  | Typicality | Plausibility | Absurdness | Taking Seriously | Arousal | Valence | Difficulty |
| --- | --- | --- | --- | --- | --- | --- | --- |
| Judgment | .722^**^ | .722^**^ | -.596^**^ | .437^*^ | .269 | .169 | -.201 |
| Typicality |  | .582^**^ | -.893^**^ | .730^**^ | .283 | .345 | -.357 |
| Plausibility |  | - | -.483^*^ | .572^**^ | .595^**^ | -.244 | .072 |
| Absurdness |  |  | - | -.726^**^ | -.209 | -.363 | .400^*^ |
| Taking Seriously |  |  |  | - | .481^*^ | -.002 | -.092 |
| Arousal |  |  |  |  | - | -.395^*^ | .151 |
| Valence |  |  |  |  |  | - | -.607^**^ |

*Note*. Typicality, plausibility, absurdness, taking seriously: 1 = strongly oppose, 7 = strongly support; Arousal: 1 = not at all arousing, 7 = very arousing; Valence: 1 = very negative feelings, 7 = very positive feelings; Difficulty: 1 = not difficult, 7 = very difficult; Moral judgment: 1 = completely inappropriate, 7 = completely appropriate.

^*:^ *p* < .05. ^**^ *p* < .01.

**Table S6**

*Correlation Matrix for Evaluations of Factual Dilemmas where Deontology Coincides with Action (N = 12), Study 2*

|  | Typicality | Plausibility | Absurdness | Taking Seriously | Arousal | Valence | Difficulty |
| --- | --- | --- | --- | --- | --- | --- | --- |
| Judgment | .112 | -.083 | -.431 | .168 | -.402 | .593^*^ | -.074 |
| Typicality |  | .790^**^ | -.819^**^ | .717^**^ | .187 | .101 | .231 |
| Plausibility |  | - | -.774^**^ | .781^**^ | .479 | -.440 | .513 |
| Absurdness |  |  | - | -.750^**^ | -.142 | -.069 | -.328 |
| Taking Seriously |  |  |  | - | .556 | -.122 | .551 |
| Arousal |  |  |  |  | - | -.580^*^ | .748^**^ |
| Valence |  |  |  |  |  | - | -.414 |

*Note*. Typicality, plausibility, absurdness, taking seriously: 1 = strongly oppose, 7 = strongly support; Arousal: 1 = not at all arousing, 7 = very arousing; Valence: 1 = very negative feelings, 7 = very positive feelings; Difficulty: 1 = not difficult, 7 = very difficult; Moral judgment: 1 = completely inappropriate, 7 = completely appropriate.

^*:^ *p* < .05. ^**^ *p* < .01.

Recoding moral judgment so that greater values indicate more agreement with the utilitarian option, the correlation between the combined realism measure (consisting of typicality, plausibility, and reverse coded absurdness, Cronbach’s α = .88) and the utilitarianism score are depicted in Figure S2.

**Figure S2**

*Correlation Between Realism Evaluations and Moral Judgments for Dilemma Types Separately, Study 2*


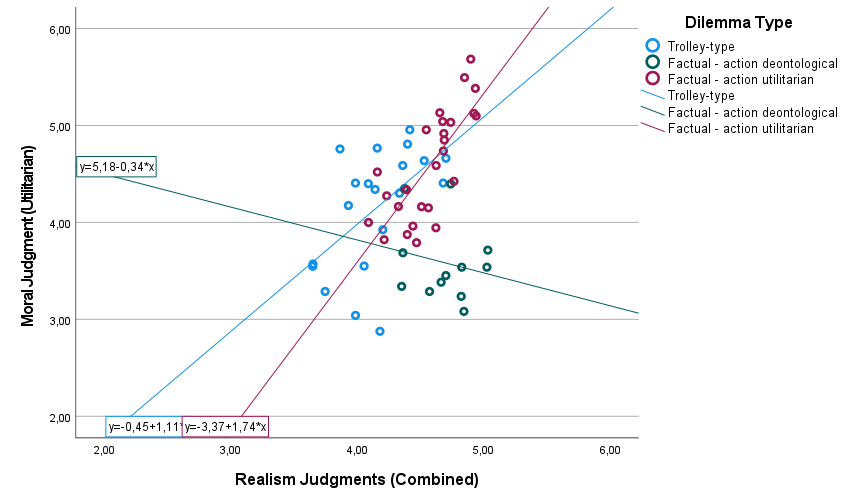


*Note*. Higher moral judgment values indicate more utilitarian judgments. Both scales ranged from 1 to 7.
